# Sonogenetics Nanomedicine Activates the cGAS‐STING Pathway for Immunotherapy of Triple‐Negative Breast Cancer

**DOI:** 10.1002/advs.77817

**Published:** 2026-09-16

**Authors:** Junya Song, Yan Liu, Shifeng Zhao, Cheng Zhu, Hongchang Guo, Jin Chang, Jun Kang

**Affiliations:** ^1^ School of Life Sciences Faculty of Medicine Tianjin University Tianjin China; ^2^ Department of Cardiovascular Surgery Beijing Anzhen Hospital Capital Medical University Beijing China

**Keywords:** cancer immunotherapy, cGAS‐STING signaling pathway, human epidermal growth factor receptor 3, sonogenetics

## Abstract

Due to pronounced tumor heterogeneity and the lack of effective therapeutic options, triple‐negative breast cancer (TNBC) remains a formidable clinical challenge for the development of effective medicines. Here, we report a HER3‐targeted sonogenetic mechano‐immunomodulatory nanoplatform (HSMIN‐LNP), achieving integrated synergistic therapy for TNBC. HSMIN‐LNP employs a de novo AI‐designed HER3‐targeting miniprotein (HTIM) and co‐delivers the TRPV4 (mechanosensory) plasmid together with an NFAT‐promoted IL‐15 gene. The results demonstrated that HTIM directly bound and suppressed HER3 signaling, inhibiting the downstream PI3K/AKT/mTOR pathway. This process markedly impaired tumor cell migratory capacity. The NPs also exhibited a uniform and stable nanostructure with high plasmid encapsulation efficiency and enabled ultrasound (US)‐triggered local IL‐15 induction through TRPV4‐mediated Ca^2+^/NFAT signaling. Mechanistically, US‐activated TRPV4 further elicited pronounced Ca^2+^ overload, reactive oxygen species accumulation, mitochondrial damage, and apoptosis in tumor cells. It simultaneously activated cGAS‐STING‐dependent inflammatory and interferon signaling pathways, thereby contributing to tumor cell death and immune‐associated antitumor responses. In vivo studies further verified that HSMIN‐LNP significantly enhanced dendritic cell maturation, NK cell and CD8^+^ T cell responses, as well as memory T cell formation. Collectively, this study provides a promising avenue for integrated precision therapy of TNBC.

## Graphical abstract

A HER3‐targeted sonogenetic lipid nanoparticle platform is developed for triple‐negative breast cancer therapy. An AI‐designed miniprotein inhibits HER3 signaling, while TRPV4 and NFAT‐responsive IL‐15 plasmids enable ultrasound‐controlled calcium signaling and cytokine expression. The system induces Ca^2+^ overload, ROS generation, mitochondrial damage, and cGAS‐STING activation, promoting tumor apoptosis and enhancing antitumor immunity.

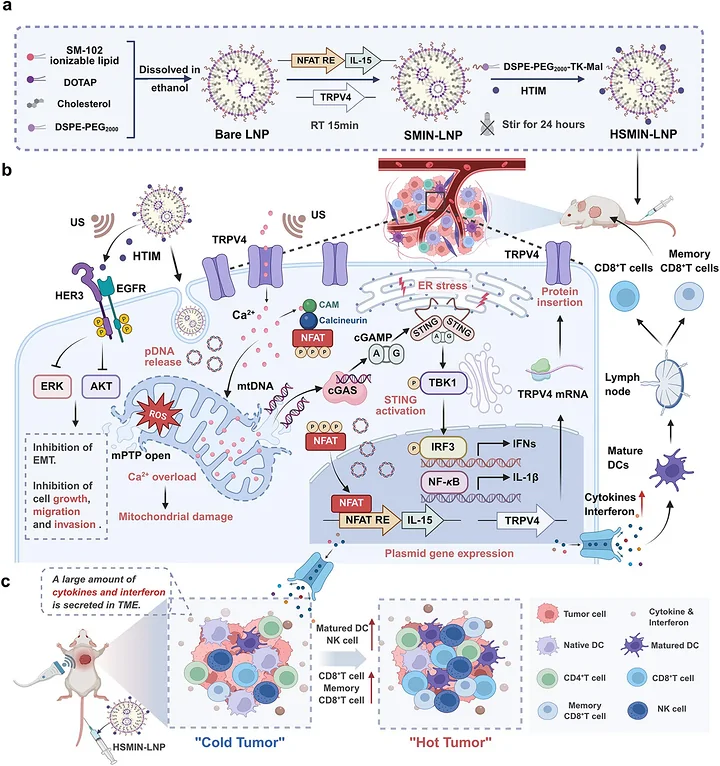

## 1 Introduction

Triple‐negative breast cancer (TNBC) accounts for ∼10%–15% of breast cancers (BC). It is defined by the absence of estrogen receptor, progesterone receptor, and human epidermal growth factor receptor 2 (HER2) expression [1]. TNBC is characterized by pronounced molecular heterogeneity and aggressive biological behavior, and is associated with a high risk of early recurrence and distant metastasis, ultimately leading to poor overall prognosis [2]. Although immune checkpoint inhibitors (ICIs) and antibody‐drug conjugates (ADCs) have provided new therapeutic options for a subset of patients, chemotherapy remains the cornerstone of systemic treatment for most patients with TNBC [3, 4]. Therefore, the identification of therapeutically relevant molecular targets and the development of more effective treatment strategies remain major priorities in TNBC research.

In recent years, human epidermal growth factor receptor 3 (HER3/ErbB3) has been increasingly recognized as a critical driver of tumor progression, metastasis, and therapeutic resistance, and has thus emerged as an important target in BC research [5]. HER3 can form heterodimers with HER2 or epidermal growth factor receptor (EGFR), recruiting phosphoinositide 3‐kinases (PI3K) and activating downstream PI3K/AKT signaling pathways, ultimately promoting tumor cell proliferation, migration, and survival [6, 7]. Consistent with this, suppression of the PI3K/AKT axis has been associated with antitumor activity [8]. As a principal HER3 ligand, neuregulin‐1β (NRG1β) can initiate ligand‐dependent HER3 activation and facilitate the formation of heterodimers, thereby linking extracellular growth factor stimulation to aggressive phenotypes and therapeutic resistance [9]. In TNBC, the HER3‐ and EGFR‐coordinated signaling network has been closely associated with an increased risk of metastasis and poorer survival outcomes [10]. Advances in understanding the etiological roles of HER family members have promoted the transition of HER3‐targeted research from mechanistic investigation toward therapeutic development. Although HER3‐targeting monoclonal antibodies, including lumretuzumab and seribantumab, have shown preliminary activity in early clinical studies, their clinical application has been hindered by modest efficacy and an ill‐defined beneficiary population [11].

Cancer immunotherapy has become an integral component of treatment for solid tumors by enhancing effector T cell activity. It can remodel the immunosuppressive microenvironment and induce durable immune memory [12, 13]. Cytokine therapy approaches can rapidly amplify T cell‐ and NK cell‐mediated antitumor responses and are widely used to potentiate immunotherapeutic efficacy [14]. However, free cytokines are often limited by a short in vivo half‐life, high systemic exposure, and a narrow therapeutic window, while sustained overexpression may provoke severe inflammatory toxicity or even cytokine storm. Consequently, recent efforts have increasingly shifted toward localized and controllable delivery strategies [14, 15]. Lipid nanoparticles (LNPs) encompassing mRNA, siRNA, and plasmid DNA have been widely adopted for on‐site expression and secretion of cytokines and functional proteins [16, 17]. Further optimization of ionizable lipid composition, lipid ratios, and surface decoration could enhance tumor accumulation, cellular uptake, and endosomal escape [18, 19].

To avoid uncontrolled immune activation, exogenous regulatory modalities have been introduced to achieve spatiotemporal control of transgene expression and immune responses. Ultrasound (US) has been extensively explored in clinical settings because of its noninvasive nature and deep tissue penetration [20, 21, 22]. In recent years, sonogenetics has expanded from neural modulation to precision cancer therapy. Its central principle is the introduction of mechanosensitive ion channels or US‐responsive genetic circuits that enable cells to undergo Ca^2+^ influx or initiate transgene expression only under a predefined acoustic field, thereby allowing precise control of antitumor immune responses [23, 24, 25]. Low‐intensity pulsed US (LIPUS), which exhibits favorable tissue safety, can provide repeated and noninvasive stimulation in deep tissues and has been used to activate multiple mechanosensitive ion channels [26, 27]. In previous work, we reported that US activation of the large‐conductance mechanosensitive ion channel MscL on tumor cell membranes induced sustained calcium overload and tumor cell death, thereby suppressing tumor growth in vivo [28]. However, therapeutic strategies capable of precisely and controllably activating the tumor immune microenvironment to achieve effective treatment of TNBC remain largely lacking.

**FIGURE 1:**
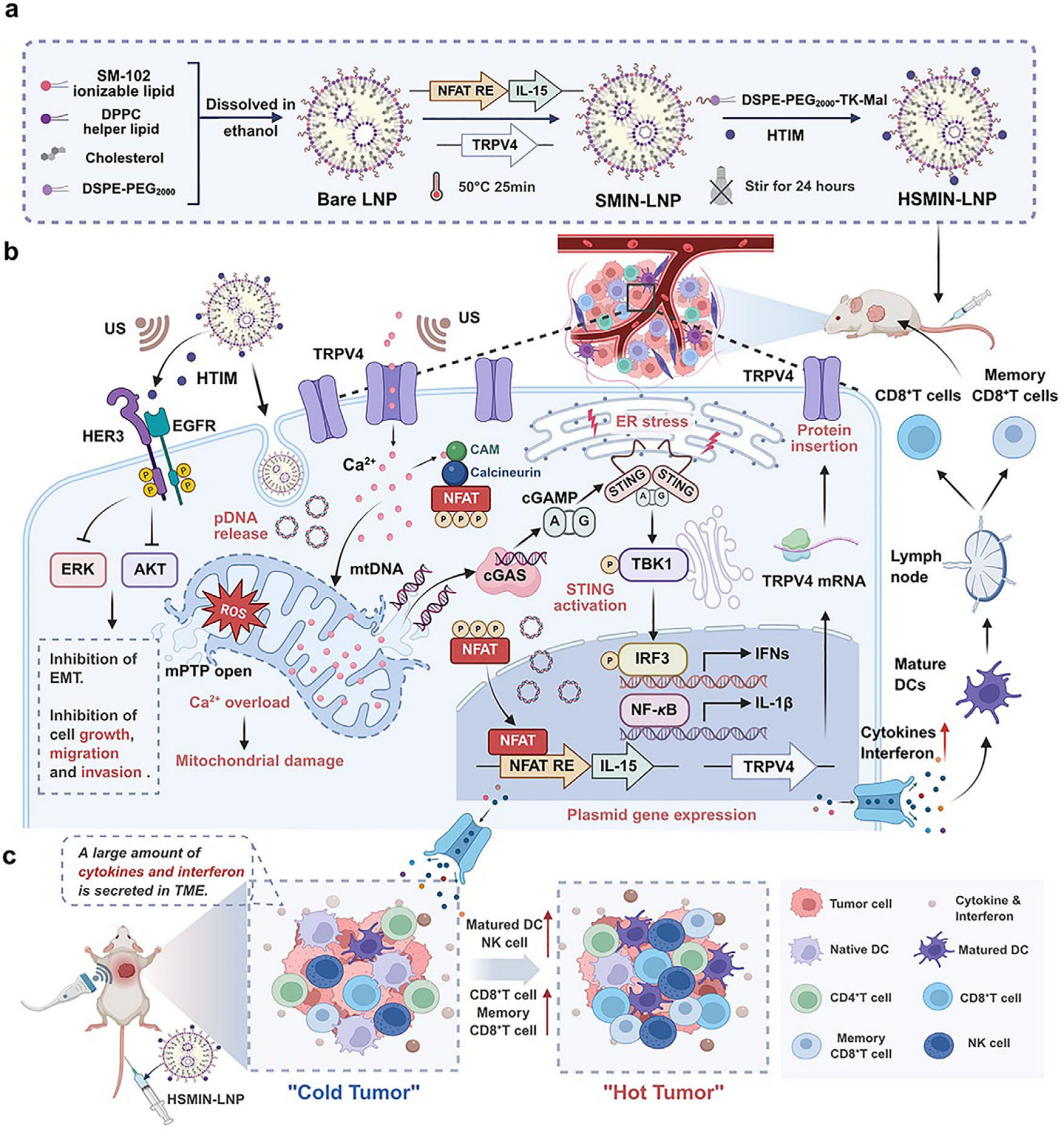
Schematic illustration of the synthesis of HSMIN‐LNP and its mechanism of action in sonogenetic immunotherapy against TNBC.

In this work, we developed HSMIN‐LNP for the precise treatment of TNBC (Figure 1). By decorating the surface of lipid nanoparticles with a de novo AI‐designed HER3‐targeting inhibitory miniprotein (HTIM), the nanoplatform was endowed with active tumor recognition and enabled targeted disruption of signaling pathways associated with tumor proliferation and migration. In parallel, it co‐delivered a transient receptor potential vanilloid 4 (TRPV4) expression plasmid and a nuclear factor of activated T‐cells (NFAT)‐responsive IL‐15 expression construct to establish a US‐controllable, Ca^2+^‐dependent gene regulatory module. Through Ca^2+^ influx triggered by activation of the mechanosensitive ion channel and the subsequent engagement of the NFAT transcriptional program, this study aimed to achieve localized inducible expression of IL‐15 and further evaluate its synergistic roles in calcium‐overload‐mediated tumor cell killing and antitumor immune activation. Overall, this nanoplatform is expected to integrate the complementary advantages of molecular targeting, sonogenetic regulation, and immune activation, thereby offering a new strategy for the integrated treatment of TNBC.

## 2 Results and Discussion

### 2.1 De Novo AI‐Designed HTIM Inhibits HER3 Signaling and Migration in 4T1 Cells

**FIGURE 2:**
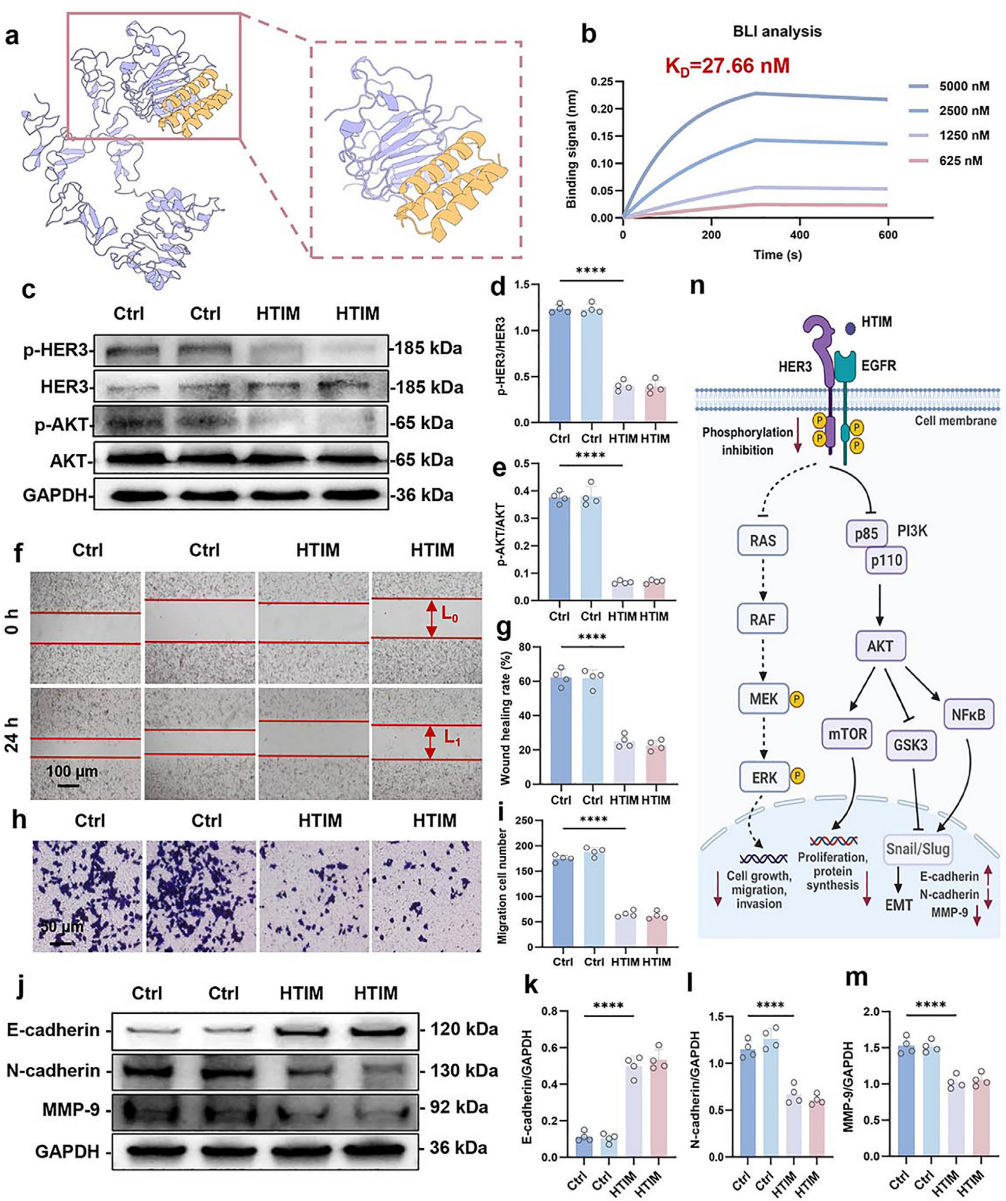
AI‐designed HER3‐targeting miniprotein inhibits HER3 signaling and suppresses migration in 4T1 cells. (a) AlphaFold3‐predicted structure of the HER3 extracellular domain (purple) bound to the designed HTIM (orange), showing the predicted interaction interface. (b) BLI‐based kinetic characterization of HTIM binding to recombinant HER3. (c–e) Western blot analysis of phosphorylation levels of HER3 and AKT in 4T1 cells treated with HTIM. (f, g) Representative results of wound‐healing assays in 4T1 cells after HTIM treatment. Scale bar: 100 µm. (h, i) The results of the Transwell assay in 4T1 cells after HTIM treatment. Scale bar: 50 µm. (j–m) Western blot analysis of E‐cadherin, N‐cadherin, and MMP‐9 in 4T1 cells treated with HTIM. (n) Working model depicting HTIM‐mediated blockade of HER3 activation and downstream signaling pathways linked to migration. Data are presented as mean ± SD (n = 4). The *p* values were calculated using one‐way ANOVA; ns: not significant, ^*^ *p* < 0.05, ^**^ *p* < 0.01, ^***^ *p* < 0.001, ^****^ *p* < 0.0001.

By analyzing the structural mechanism of NRG1β recognition and its conformational change toward HER3‐EGFR pro‐oncogenic heterocomplex [9, 29], we identified the HER3 extracellular domain I as a computational protein design target to develop miniproteins that recognize domain I and disrupt HER3/NRG1β interfaces. We applied the deep learning models RFdiffusion and ProteinMPNN to design miniproteins (molecular weights of 8–12 kDa), express them, and screen their binding affinities toward HER3 (Figure S1a–d). The biolayer interferometry (BLI) experiment indicated that the strongest binding event (Miniprotein‐1, hereafter referred to as HTIM) featured an equilibrium dissociation constant (K_D_) of 27.66 nM (Figure 2a,b). The HTIM/HER3 interaction exhibited rapid association and slow dissociation kinetics, suggesting a prolonged receptor residence time for HTIM, which may favor more stable and durable target occupancy in vivo.

We next investigated whether HTIM could sustain target occupancy and effectively block receptor‐proximal signaling in a cellular context. In TNBC 4T1 cells, where HER2 is absent or expressed at low levels, phosphorylation‐dependent activation of HER3 typically relies on heterodimerization with kinase‐competent receptors such as EGFR, thereby driving downstream signaling through the PI3K‐AKT‐mTOR and RAS‐RAF‐MEK‐ERK pathways and further regulating cytoskeletal remodeling and epithelial‐mesenchymal transition (EMT)‐associated molecular programs linked to migration and invasion [5, 30, 31]. We therefore first examined the activation status of HER3 and its key downstream signaling nodes in 4T1 cells, a mouse TNBC cell line. Western blot analysis showed that HTIM treatment markedly reduced HER3 phosphorylation. Consistent with this, the phosphorylation levels of AKT, mTOR, and ERK were also substantially decreased, indicating that HTIM systematically attenuated HER3‐driven pro‐migratory and pro‐proliferative signaling output without altering total protein expression levels (Figure 2c–e and Figure S2a–c).

Following molecular validation of HER3 pathway inhibition, we next assessed its effect on cell migration. In wound‐healing assays, the control group exhibited a wound closure rate of 61.6% at 24 h, whereas HTIM‐treated cells showed a markedly reduced closure rate of 23.6%, representing a ∼62% decrease in migratory capacity (Figure 2f,g). Consistently, Transwell assays showed that, within the same field of view, the average number of migrated cells decreased from approximately 181 in the control group to 64 following HTIM treatment, representing a reduction of approximately 65% (Figure 2h,i). Together, these results indicate that inhibition of HER3‐proximal signaling by HTIM is effectively translated into a substantial impairment of the migratory capacity of 4T1 cells.

To further delineate the phenotypic basis underlying this antimigratory effect, we examined multiple markers associated with metastatic potential. HTIM treatment significantly upregulated the epithelial marker E‐cadherin, while downregulating the mesenchymal marker N‐cadherin and the matrix‐remodeling factor MMP‐9 (Figure 2j–m). These findings suggest that HTIM partially reverses the mesenchymal phenotype of 4T1 cells, thereby reducing the loss of cell‐cell adhesion and extracellular matrix degradation potential, ultimately suppressing the migratory phenotype of tumor cells.

Taken together, these findings indicate that HTIM interacts with the extracellular domain of HER3 and attenuates HER3‐mediated pro‐migratory signaling in 4T1 cells. This effect is accompanied by reduced HER3‐AKT pathway activation, impaired cell migration, and suppression of EMT‐associated phenotypic maintenance, ultimately limiting the migratory potential of triple‐negative breast cancer cells (Figure 2n).

### 2.2 Preparation and Characterization of the Sonogenetic Nanoparticle HSMIN‐LNP

**FIGURE 3:**
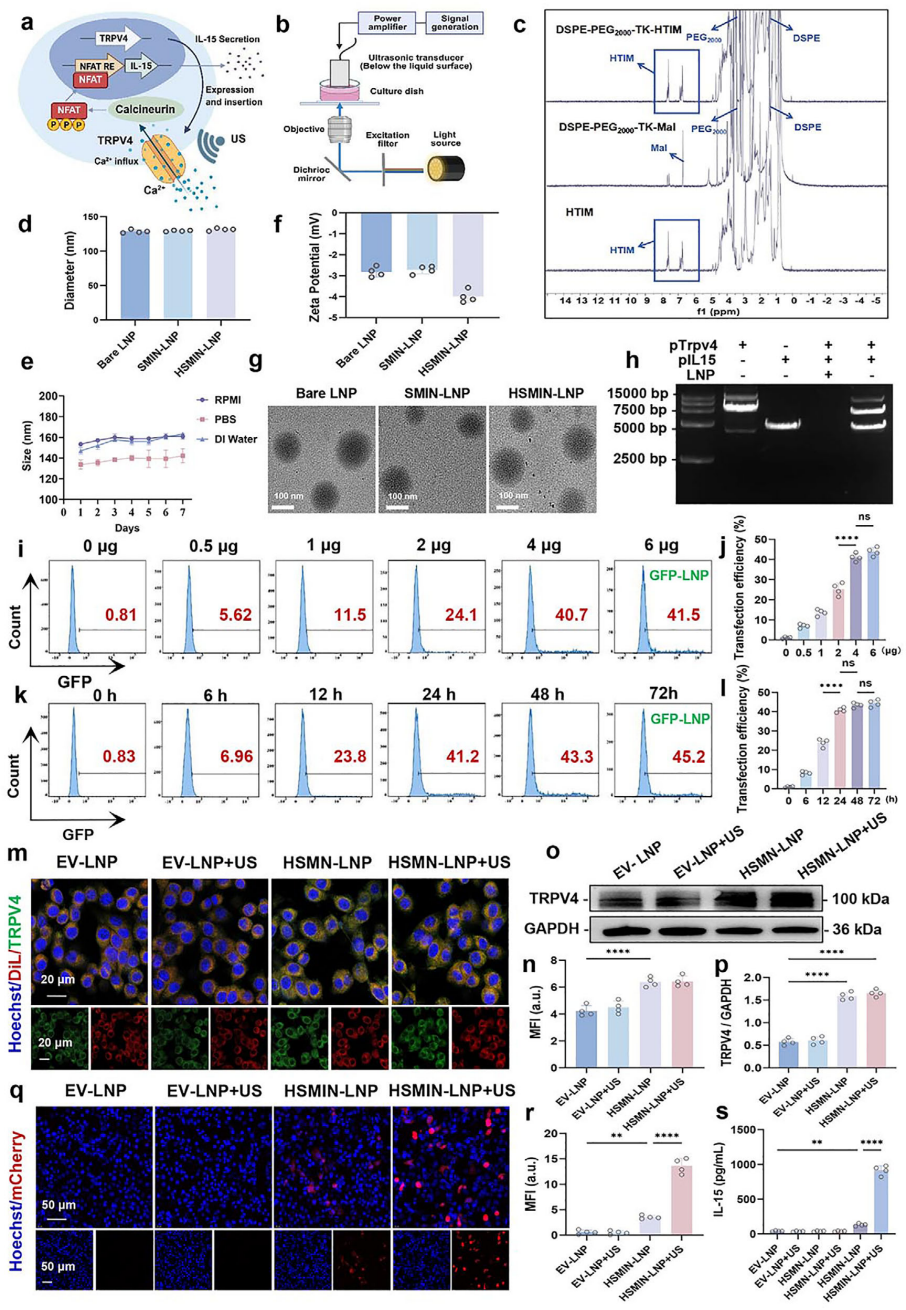
Preparation and characterization of HSMIN‐LNP. (a) Schematic illustration of US‐induced activation of the mechanosensitive ion channel TRPV4, leading to plasmid gene expression. (b) Schematic of the US stimulation and fluorescence imaging setup. The ultrasonic transducer is driven by an arbitrary waveform generator through a power amplifier to deliver LIPUS to cells in a culture dish, while fluorescence imaging is performed simultaneously through the optical path shown. (c) ^1^H NMR spectra of DSPE‐PEG_2000_‐TK‐Mal, HTIM, and DSPE‐PEG_2000_‐TK‐HTIM in deuterium oxide (D_2_O). (d) Size distribution of Bare LNP, SMIN‐LNP, and HSMIN‐LNP by DLS. (e) Hydrodynamic diameters of HSMIN‐LNP in deionized water, PBS, and RPMI 1640 for 7 days. (f) Zeta potential of Bare LNP, SMIN‐LNP, and HSMIN‐LNP. (g) Representative TEM images of Bare LNP, SMIN‐LNP, and HSMIN‐LNP. Scale bars: 100 nm. (h) Agarose gel analysis of plasmid‐loaded LNP. (i–l) Transfection of *pCMV‐GFP* plasmid in 4T1 cells mediated by Bare LNP. 4T1 cells were seeded in 12‐well plates and transfected with different amounts of plasmid. (i) Representative flow cytometry histograms of GFP expression 72 h after transfection with increasing plasmid doses (0–6 µg per well). (j) Quantification of transfection efficiency based on the percentage of GFP‐positive cells shown in (i). To evaluate the time‐dependent expression, cells were transfected with 4 µg of plasmid per well and analyzed at different time points. (k) Representative flow cytometry histograms of GFP expression at 0, 6, 12, 24, 48, and 72 h post‐transfection. (l) Corresponding quantification of transfection efficiency derived from the percentage of GFP‐positive cells shown in (k). (m) Representative confocal images showing HSMN‐LNP treatment increased the expression level of TRPV4 protein in 4T1 cells. TRPV4 protein (green: FITC‐conjugated secondary antibodies), cell membrane (red: Dil), and nuclei (blue: DAPI). Scale bar: 20 µm. (n) Analysis of the fluorescence intensity changes of the TRPV4 protein. (o‐p) Western blot analysis of TRPV4 protein expression after transfection with pTrpv4 plasmid using HSMN‐LNP. (q) Representative confocal images showing that the red fluorescence intensity significantly increased in 4T1 cells transfected with HSMIN‐LNP (pIL15 plasmid carrying an mCherry fluorescent reporter) after US stimulation. mCherry (red) and nuclei (blue: Hoechst). Scale bar: 50 µm. (r) Quantification of mCherry mean fluorescence intensity corresponding to (q), showing changes in US‐evoked mCherry expression level across indicated conditions. (s) ELISA analysis of IL‐15 secretion in the culture supernatant after transfection and US treatment. Data are presented as mean ± SD (n = 4). The *p* values were calculated using one‐way ANOVA; ns: not significant, ^*^ *p* < 0.05, ^**^ *p* < 0.01, ^***^ *p* < 0.001, ^****^ *p* < 0.0001.

We next sought to integrate targeted inhibition with controllable immune activation. The sonogenetic nanoparticle system, HSMIN‐LNP, was constructed. As illustrated in Figure 3a, HSMIN‐LNP co‐encapsulated the TRPV4 plasmid (*pECMV‐Trpv4‐m‐FLAG*, denoted as pTrpv4) and the NFAT‐responsive proinflammatory cytokine IL‐15 plasmid (*pGL4.30[NFAT‐RE‐IL15‐Hygro]*, denoted as pIL15). Upon US stimulation, the TRPV4 channel is activated. This induces Ca^2+^ influx, activating calcineurin and subsequently promoting NFAT dephosphorylation and nuclear translocation, thereby driving IL‐15 expression [32]. Notably, the IL‐15 expression cassette contains three NFAT‐response elements (RE) upstream of the promoter, indicating a calcium‐threshold‐dependent activation mechanism in which IL‐15 expression and secretion are initiated only when intracellular Ca^2+^ influx exceeds a critical threshold. Meanwhile, HTIM was conjugated to the nanoparticle surface through a thioketal (TK) linker. Under US exposure, cavitation, localized thermal effects, and reactive oxygen species (ROS) generation collectively promoted cleavage of the TK linker, thereby enabling controlled release of HTIM [33, 34]. Through this design, HSMIN‐LNP establishes a dual‐module regulatory mechanism integrating intracellular transgene expression with extracellular receptor blockade. Furthermore, the US stimulation was delivered by a transducer system driven by a signal generator and power amplifier (Figure 3b), providing a reproducible exogenous triggering condition for subsequent nanoparticle construction and functional validation. In this study, the LIPUS regimen was designed to activate the TRPV4‐based mechanotransduction module through repeated but non‐continuous acoustic stimulation. The 1 MHz carrier frequency was selected to provide effective mechanical perturbation at the cell membrane within a commonly used low‐MHz ultrasound range, while the 1 kHz pulse repetition frequency and 20% duty cycle enabled intermittent stimulation, which may facilitate temporal integration of Ca^2+^ signals while avoiding continuous mechanical exposure.

To enable covalent conjugation of HTIM to the lipid moiety, a cysteine residue was introduced at its C terminus, allowing the thiol group to undergo a Michael addition reaction with the maleimide (Mal) group in DSPE‐PEG_2000_‐TK‐Mal, thereby forming a stable thioether bond [35]. ^1^H nuclear magnetic resonance (NMR) spectra of the conjugate showed the appearance of characteristic HTIM‐associated peaks together with the disappearance of the Mal signal, indicating successful coupling and confirming the formation of DSPE‐PEG_2000_‐TK‐HTIM (Figure 3c), which was subsequently used as a key component for the synthesis of the sonogenetic nanoparticles.

The nanoparticles were then prepared by rapid mixing of the lipid components with the plasmid formulation. Dynamic light scattering (DLS) analysis showed that the mean hydrodynamic diameters of bare LNPs, LNPs co‐loaded with TRPV4 and IL‐15 plasmids (sonogenetic mechano‐immunomodulatory nanoplatform, SMIN‐LNP), and HTIM‐decorated LNPs (HSMIN‐LNP) were all maintained at ∼130 nm, with no significant differences among the groups (Figure 3d), indicating that neither plasmid encapsulation nor HTIM surface functionalization markedly altered particle size. Consistently, longitudinal monitoring of HSMIN‐LNP over 7 days revealed high colloidal stability in deionized water, PBS, and RPMI 1640 medium (Figure 3e and Figure S3a). Zeta potential measurements further showed a slight increase in surface negativity after HTIM conjugation, with HSMIN‐LNP exhibiting a surface potential of ‐3.94 mV (Figure 3f).

Transmission electron microscopy (TEM) revealed that all three nanoparticle formulations displayed regular spherical or quasi‐spherical morphologies with good dispersity and well‐defined boundaries (Figure 3g). Furthermore, the plasmid encapsulation efficiency of HSMIN‐LNP remained stable over 7 days and consistently exceeded 90% (Figure S3b). In addition, to evaluate the US‐triggered release behavior of HTIM, we quantified its release using a BCA assay. HTIM was rapidly released upon US stimulation, reaching a cumulative release of approximately 80% within 8 h, after which the release profile gradually plateaued (Figure S3c). In gel retardation assays, migration of both pTrpv4 and pIL15 was markedly inhibited under LNP‐encapsulated conditions, with no evident free plasmid bands observed, further confirming efficient plasmid loading within the nanoparticles (Figure 3h). Collectively, these results demonstrate that HSMIN‐LNP retains favorable size distribution, structural integrity, and nucleic acid loading capacity while achieving successful surface functionalization.

We next assessed the transfection efficiency and US responsiveness of the synthesized nanoparticles in 4T1 cells. To optimize transfection conditions, Bare LNPs were first used to deliver pCMV‐GFP. The percentage of GFP‐positive cells increased in a dose‐ and time‐dependent manner, reaching a near‐plateau at 4 µg per well in 12‐well plates and stabilizing at approximately 24 h (Figure 3i–l). These conditions were therefore selected for subsequent experiments. Under these optimized conditions, compared with LNPs loaded with an empty vector (pECMV‐MCS‐FLAG; EV‐LNP), HTIM‐functionalized nanoparticles loaded with pTrpv4 (HER3‐targeted sonogenetic mechanotransduction nanoplatform, HSMN‐LNP) markedly increased TRPV4 protein expression in 4T1 cells. Confocal imaging further showed clear colocalization of the TRPV4 signal with the plasma membrane marker, indicating successful ectopic expression of TRPV4 and its proper membrane localization (Figure 3m–p). Then, pTrpv4 and pIL15‐mCherry were co‐delivered to further evaluate US‐responsive transgene activation. A pronounced increase in mCherry fluorescence was observed only upon US stimulation, and ELISA analysis consistently confirmed a concomitant increase in IL‐15 secretion in the culture supernatant (Figure 3q–s). These results demonstrate that HSMIN‐LNP enables TRPV4‐mediated, US‐responsive IL‐15 expression and secretion in 4T1 cells.

### 2.3 Ultrasound‐Triggered Ca^2+^ Overload and ROS Amplification Drive Apoptosis in 4T1 Cells

**FIGURE 4:**
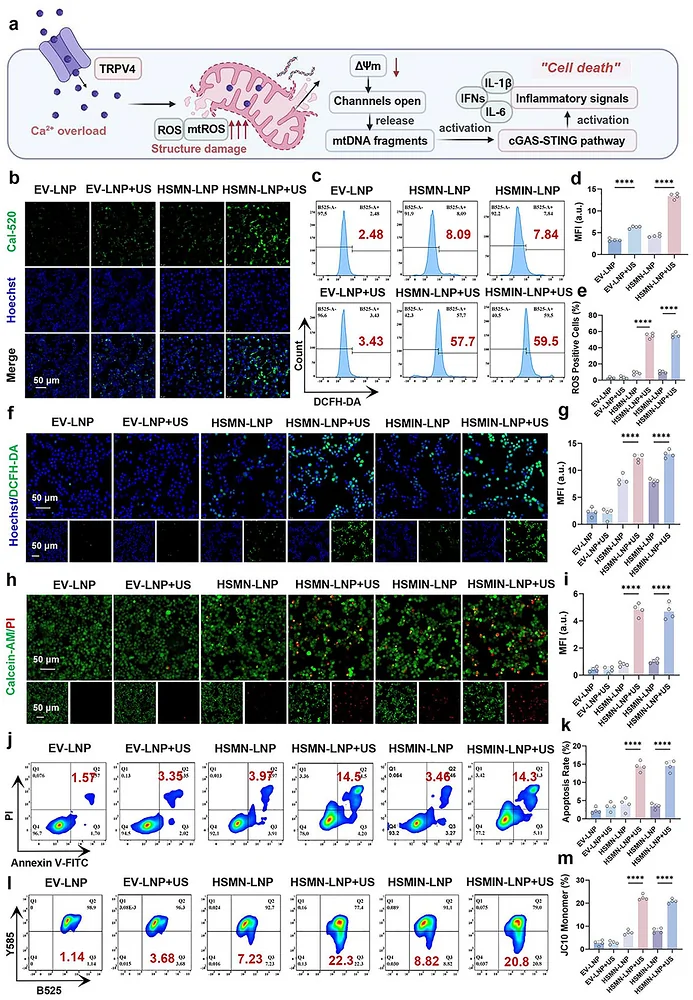
Ultrasound‐triggered Ca^2+^ overload drives ROS accumulation, mitochondrial depolarization, and apoptosis in 4T1 cells. (a) Schematic illustration of TRPV4‐mediated Ca^2+^ overload‐induced mitochondrial damage and cell death. (b) Representative confocal images of intracellular Ca^2+^ signals in 4T1 cells after treatment with EV‐LNP or HSMN‐LNP, with or without US stimulation. Ca^2+^ (green: Cal‐520) and nuclei (blue: Hoechst). Scale bar: 50 µm. (c) Flow cytometric analysis of intracellular ROS levels in 4T1 cells measured by DCFH‐DA following treatment with EV‐LNP, HSMN‐LNP, or HSMIN‐LNP, with or without US stimulation. (d) Quantification of Cal‐520 mean fluorescence intensity corresponding to (b), showing US‐evoked Ca^2+^ signal changes across indicated conditions. (e) Quantification of ROS‐positive 4T1 cells corresponding to (c). (f) Representative confocal images of intracellular ROS accumulation in 4T1 cells under the indicated treatments. ROS (green: DCFH‐DA) and nuclei (blue: Hoechst). Scale bar: 50 µm. (g) Quantification of ROS‐associated fluorescence intensity corresponding to (f). (h) Representative live/dead staining images of 4T1 cells after the indicated treatments. Live cells (green: Calcein‐AM) and dead cells (red: PI). Scale bar: 50 µm. (i) Quantification of propidium iodide–associated fluorescence intensity corresponding to (h), reflecting cell death levels. (j) Flow cytometric analysis of apoptosis in 4T1 cells by Annexin V‐FITC/PI staining after the indicated treatments. (k) Quantification of apoptotic 4T1 cells corresponding to (j). (l) Flow cytometric analysis of mitochondrial membrane potential in 4T1 cells by JC‐10 staining following the indicated treatments. (m) Quantification of JC‐10 monomer‐positive cells corresponding to (l). Data are presented as mean ± SD (n = 4). The *p* values were calculated using one‐way ANOVA; ns: not significant, ^*^ *p* < 0.05, ^**^ *p* < 0.01, ^***^ *p* < 0.001, ^****^ *p* < 0.0001.

We next investigated whether HSMIN‐LNP could convert TRPV4‐mediated mechanosensitive signaling into a canonical tumoricidal axis characterized by Ca^2+^ overload, oxidative stress, and apoptosis (Figure 4a). To this end, we first performed live‐cell imaging of intracellular Ca^2+^ dynamics using the fluorescent calcium indicator Cal‐520. As a highly sensitive Ca^2+^ probe, Cal‐520 exhibits increased fluorescence intensity in response to rising intracellular free Ca^2+^ levels, thereby enabling real‐time monitoring of calcium signaling following US stimulation [36]. To exclude potential interference from intracellular calcium store release, we first conducted control experiments in the absence of extracellular Ca^2+^. We found that US stimulation failed to further enhance the intracellular Cal‐520 fluorescence signal in cells treated with either EV‐LNP or HSMN‐LNP (Figure S4a,b), indicating that, in the absence of extracellular Ca^2+^ influx, US could not induce a detectable elevation in intracellular Ca^2+^.

Subsequently, the extracellular buffer was replaced with Ca^2+^‐containing Hanks’ balanced salt solution (HBSS) for continuous live‐cell imaging without changing the imaging field, allowing reliable comparison of US‐induced Ca^2+^ influx under different treatment conditions. In EV‐LNP treated cells, intracellular Ca^2+^ signals remained at an overall low level, but showed a modest increase upon US stimulation, indicating that US alone could induce basal Ca^2+^ entry. Notably, delivery of the pTrpv4 plasmid markedly amplified this calcium response. Following US exposure, the increase in Ca^2+^ fluorescence intensity was substantially greater in pTrpv4‐transfected cells than in the control group, and the mean fluorescence intensity under the combined transfection plus US condition was approximately threefold higher than that in the non‐US group, displaying a more pronounced calcium‐loading phenotype (Figure 4b,d).

On this basis, we next investigated whether calcium loading could amplify oxidative stress and drive changes in cell fate. Intracellular ROS levels were measured using the fluorescent probe 2′,7′‐dichlorodihydrofluorescein diacetate (DCFH‐DA). Upon oxidation by ROS, this probe is converted into the highly fluorescent product dichlorofluorescein (DCF); thus, increased fluorescence is generally indicative of elevated oxidative stress. Flow cytometric analysis showed that the proportions of ROS‐positive cells in the EV‐LNP group and the corresponding US‐treated group remained low, at approximately 2.48% and 3.43%, respectively. In contrast, within the HSMN‐LNP and HSMIN‐LNP systems, US triggering increased the proportion of ROS‐positive cells from approximately 8% to 57%–60% (Figure 4c,e). Consistent with the flow cytometry results, both confocal imaging and quantitative flow analysis demonstrated that the combination of transfection and US markedly enhanced intracellular ROS accumulation (Figure 4f,g), indicating that calcium overload is accompanied by a robust oxidative stress burst.

To further determine whether this calcium overload and oxidative stress ultimately translate into cytotoxicity, we assessed cell death and apoptosis using Calcein‐AM/PI live/dead staining and Annexin V/PI flow cytometry. Calcein‐AM labels viable cells with intact plasma membranes through esterase‐dependent green fluorescence, whereas PI selectively stains membrane‐compromised or dead cells by intercalating with nucleic acids and emitting red fluorescence. Live/dead staining revealed a marked increase in death signals following the combined transfection and US treatment. In particular, PI fluorescence reached the highest levels in the HSMN‐LNP + US and HSMIN‐LNP + US groups, showing an approximately 5.2‐fold increase relative to the control group (Figure 4h,i), indicative of substantial loss of membrane integrity and a pronounced decline in cell viability. Consistent with this, Annexin V/PI analysis confirmed a parallel increase in apoptosis in 4T1 cells. The proportion of late apoptotic cells increased from 1.57% in the control group to 14.3% in the HSMIN‐LNP + US group, representing an approximately ninefold elevation (Figure 4j,k). Collectively, these results demonstrate that TRPV4‐mediated US responsiveness induces pronounced Ca^2+^ overload and amplifies ROS accumulation in 4T1 cells, ultimately driving cell death and apoptosis. These findings establish that this sonogenetic nanoplatform can efficiently convert an exogenous physical stimulus into potent tumoricidal effects at the cellular level.

### 2.4 The Sonogenetic Nanoplatform Induces Mitochondrial Damage and Activates cGAS‐STING Signaling

The preceding results showed that TRPV4‐mediated US stimulation triggered pronounced Ca^2+^ overload in 4T1 cells and further amplified ROS accumulation and apoptosis. Given that mitochondria serve as a central hub coupling Ca^2+^ dyshomeostasis to oxidative stress [37], we hypothesized that sonogenetically induced calcium overload could be amplified at the mitochondrial level and subsequently converted into broader damage and inflammatory signaling. On this basis, we first assessed changes in mitochondrial membrane potential (MMP) in 4T1 cells using JC‐10 staining. Cells in the EV‐LNP and EV‐LNP + US groups largely maintained stable mitochondrial membrane potential, whereas the proportion of cells undergoing mitochondrial depolarization increased markedly following plasmid delivery combined with US stimulation. Quantitative analysis showed that the proportion of JC‐10 monomers increased from approximately 1.14% in the EV‐LNP group to 22.3% in the HSMN‐LNP + US group and 20.8% in the HSMIN‐LNP + US group, indicating that the combined treatment substantially exacerbated mitochondrial depolarization (Figure 4l,m).

**FIGURE 5:**
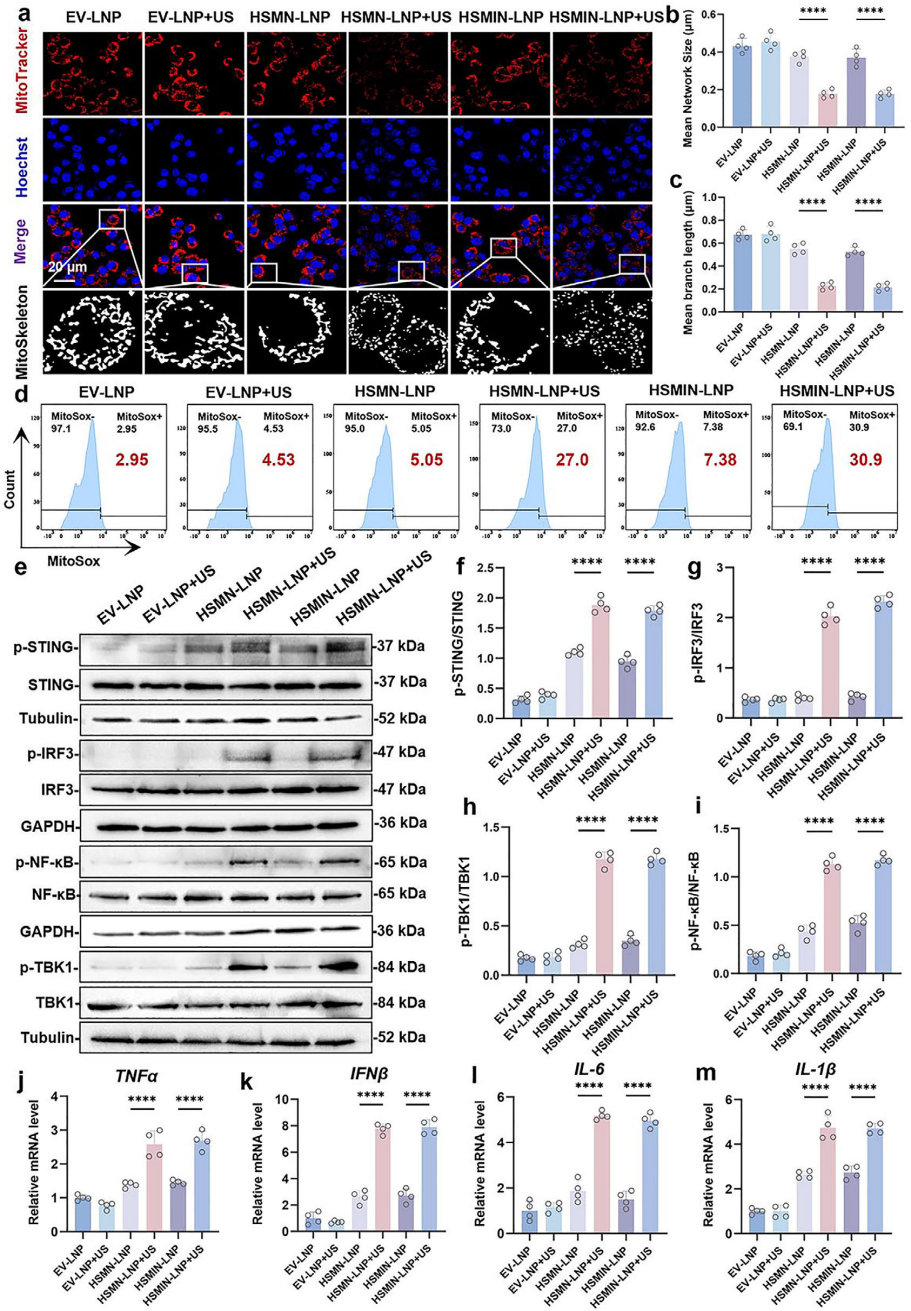
Ultrasound induces mitochondrial damage and activates cGAS‐STING signaling in 4T1 cells. (a) Representative confocal images of mitochondrial morphology in 4T1 cells stained with MitoTracker under the indicated treatments. Mitochondria (red: MitoTracker) and nuclei (blue: Hoechst). Scale bar: 20 µm. (b) Quantification of mitochondrial mean network size derived from MitoSkeleton analysis corresponding to (a). (c) Quantification of mitochondrial mean branch length corresponding to (a). (d) Flow cytometric analysis of mtROS levels in 4T1 cells measured by MitoSOX under the indicated treatments. (e) Western blot analysis of the phosphorylation levels of STING, IRF3, TBK1, and NF‐κB in 4T1 cells under the indicated treatments. (f–i) Quantification of p‐STING/STING (f), p‐IRF3/IRF3 (g), p‐TBK1/TBK1 (h), and p‐NF‐κB/NF‐κB (i) corresponding to (e). (j–m) Quantitative PCR analysis of inflammatory and interferon‐related gene expression in 4T1 cells after the indicated treatments, including *TNFα* (j), *IFNβ* (k), *IL‐6* (l), and *IL‐1β* (m). Data are presented as mean ± SD (n = 4). The *p* values were calculated using one‐way ANOVA; ns: not significant, ^*^ *p* < 0.05, ^**^ *p* < 0.01, ^***^ *p* < 0.001, ^****^ *p* < 0.0001.

Consistent with these findings, MitoTracker imaging further revealed pronounced mitochondrial morphological remodeling. As a mitochondria‐selective dye, MitoTracker enables visualization of the distribution and continuity of the mitochondrial network. Confocal imaging showed that mitochondria in the EV‐LNP, EV‐LNP + US, HSMN‐LNP, and HSMIN‐LNP groups largely retained relatively elongated, interconnected network‐like or short tubular morphologies. In contrast, mitochondria in the HSMN‐LNP + US and HSMIN‐LNP + US groups were markedly converted into shorter, more discrete punctate or rod‐like structures, with a substantial loss of network continuity, consistent with a typical fragmented phenotype (Figure 5a). Quantitative analysis further showed that the mean network size decreased from approximately 0.44 µm in the control group to 0.19 and 0.18 µm in the HSMN‐LNP + US and HSMIN‐LNP + US groups, respectively, while the mean branch length decreased from approximately 0.68 µm to 0.23 µm and 0.22 µm (Figure 5b,c), representing reductions of more than 50% in both parameters. Together, these results indicate that sonogenetically induced Ca^2+^ loading not only causes mitochondrial membrane depolarization, but is also accompanied by profound disruption of mitochondrial network integrity and structural damage.

Given the observed mitochondrial depolarization and fragmentation, we next examined mitochondrial oxidative stress using MitoSOX, a mitochondria‐targeted fluorescent probe for superoxide, whose signal intensity reflects mtROS accumulation. Flow cytometric analysis showed that the proportion of mtROS‐positive cells remained low in the EV‐LNP and EV‐LNP + US groups, at 2.95% and 4.53%, respectively. Similarly, the HSMN‐LNP and HSMIN‐LNP groups without US treatment showed only modest mtROS positivity, at 5.05% and 7.38%, respectively. By contrast, the combination of transfection and US stimulation markedly increased this signal, with mtROS‐positive cells rising to 27.0% in the HSMN‐LNP + US group and 30.9% in the HSMIN‐LNP + US group (Figure 5d). Confocal imaging yielded consistent results, further verifying pronounced mitochondrial oxidative stress under sonogenetic activation. Whereas MitoSOX fluorescence remained weak in the control and single‐treatment groups, strong and widespread red fluorescence was observed in both the HSMN‐LNP + US and HSMIN‐LNP + US groups. Quantitative analysis further showed that the MFI in the transfection‐plus‐US groups increased by approximately 3.5‐fold relative to the matched non‐US groups (Figure S5a,b).

Taken together, the loss of mitochondrial membrane potential, fragmentation of the mitochondrial network, and marked accumulation of mtROS indicate that TRPV4‐ and US‐mediated Ca^2+^ overload induces a clear mitochondrial damage phenotype. Previous studies have shown that sustained mitochondrial depolarization and oxidative stress are frequently accompanied by opening of the mitochondrial permeability transition pore (mPTP) and increased mitochondrial membrane permeability, thereby promoting the release of mitochondrial DNA (mtDNA) into the cytosol and subsequently activating the cGAS‐STING cytosolic DNA‐sensing pathway [38, 39]. On this basis, we next examined the activation status of the cGAS‐STING signaling axis. Western blot analysis showed that, compared with the EV‐LNP, EV‐LNP + US, and other single‐treatment groups, the HSMN‐LNP + US and HSMIN‐LNP + US groups exhibited significantly increased levels of p‐STING/STING, p‐TBK1/TBK1, p‐IRF3/IRF3, and p‐NF‐κB/NF‐κB (Figure 5e–i). These results indicate that the mitochondrial damage‐associated cytosolic DNA‐sensing program was effectively engaged and further propagated to downstream innate immune transcriptional signaling.

Next, whether cGAS‐STING pathway activation was sufficient enough to drive downstream inflammatory and interferon‐associated transcriptional programs was detected. qPCR analysis showed that *TNFα*, *IFNβ*, *IL‐6*, and *IL‐1β* transcripts were all markedly increased in the HSMN‐LNP + US and HSMIN‐LNP + US groups (Figure 5j–m). Notably, *IFNβ* showed the strongest induction, increasing by approximately eightfold over control levels, whereas *IL‐6* and *IL‐1β* increased by approximately fivefold, and *TNFα* by approximately 2.5‐ to 3‐fold. To further define downstream STING‐driven interferon‐stimulated and immune‐recruiting transcriptional outputs, we additionally measured *CXCL9*, *CXCL10*, and *ISG15*, all of which were significantly upregulated in the combined treatment groups (Figure S6a–c).

Taken together, these results elucidate a sequential signaling cascade triggered by sonogenetic therapy. Specifically, TRPV4‐dependent US stimulation induces a rapid influx of intracellular Ca^2+^. This pronounced Ca^2+^ overload subsequently disrupts mitochondrial homeostasis, leading to severe mitochondrial dysfunction and structural damage. Consequently, mtDNA is released into the cytosol, where it serves as a potent danger signal. The cytosolic mtDNA is then recognized by the DNA sensor cGAS, driving robust activation of the cGAS‐STING pathway. This sequential mechanism bridging Ca^2+^ overload and mtDNA‐induced cGAS‐STING signaling effectively triggers downstream inflammatory and interferon signaling programs. This mechanism provides a vital mechanistic link between sonogenetic tumor cell killing and the initiation of a broad antitumor immune response.

### 2.5 In Vivo Tumor Targeting, Pharmacokinetic Behavior, and Transgene Expression Evaluation of HSMIN‐LNP

**FIGURE 6:**
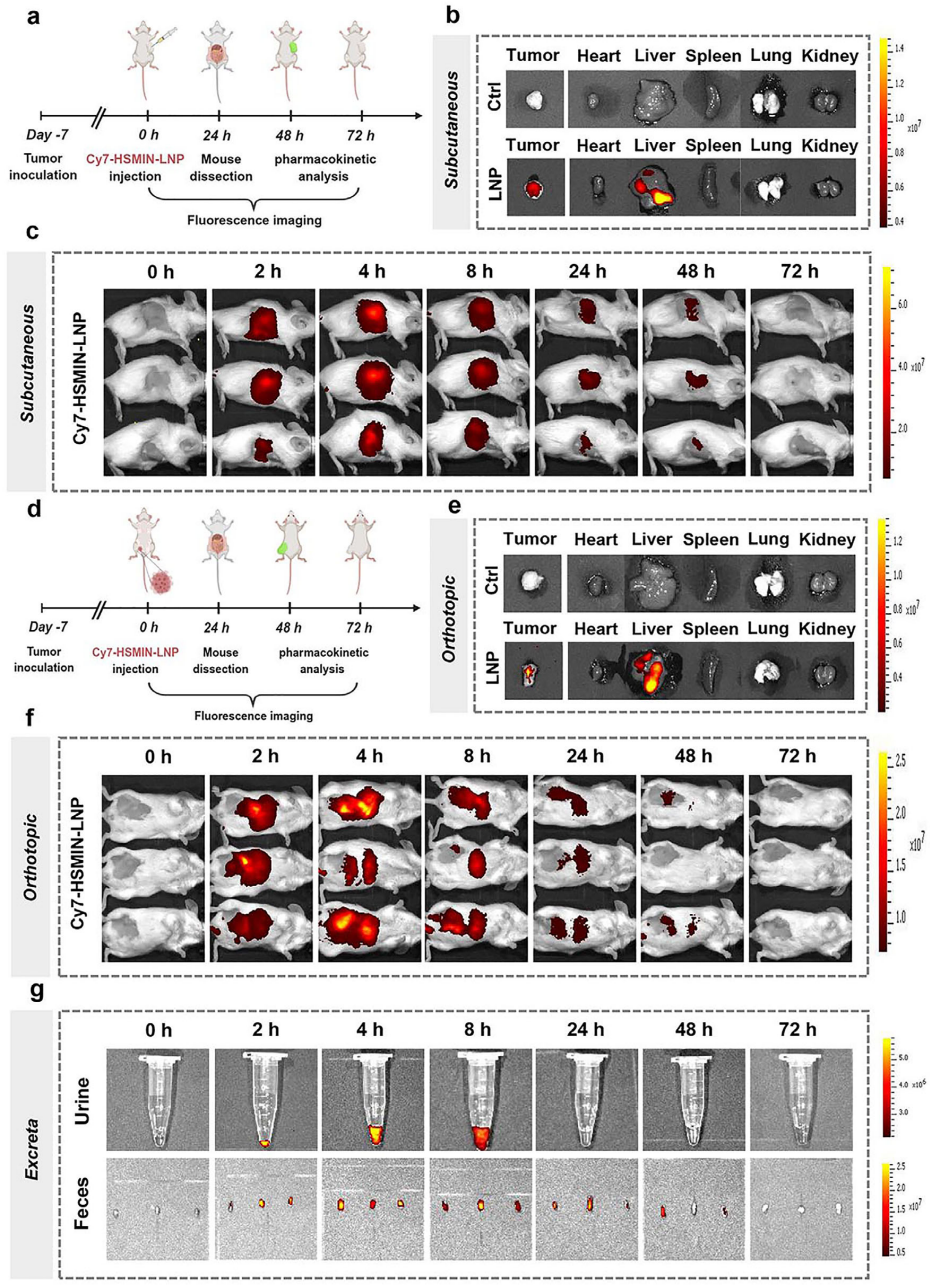
In vivo tumor‐accumulation, biodistribution, and metabolic clearance of HSMIN‐LNP in mice. (a) Schematic illustration of the in vivo fluorescence imaging workflow in the subcutaneous 4T1 tumor model. (b) Ex vivo fluorescence images of tumors and major organs harvested from subcutaneous tumor‐bearing mice at 24 h post‐injection, including tumor, heart, liver, spleen, lung, and kidney. (c) Representative in vivo fluorescence images of subcutaneous breast tumor‐bearing mice at the indicated time points (0, 2, 4, 8, 24, 48, and 72 h) after intravenous injection of Cy7‐HSMIN‐LNP. (d) Schematic illustration of the in vivo fluorescence imaging workflow in the orthotopic 4T1 tumor model. (e) Ex vivo fluorescence images of tumors and major organs collected from orthotopic tumor‐bearing mice at 24 h post‐injection, including tumor, heart, liver, spleen, lung, and kidney. (f) Representative in vivo fluorescence images of orthotopic breast tumor‐bearing mice at the indicated time points after intravenous injection of Cy7‐HSMIN‐LNP. (g) Representative fluorescence images of urine and fecal samples collected at the indicated time points after Cy7‐HSMIN‐LNP administration to assess the in vivo excretion profile.

We next systematically evaluated the in vivo tumor‐targeting behavior and metabolic profile to assess the translational feasibility of HSMIN‐LNP. As outlined in Figure 6a,d, HSMIN‐LNP was fluorescently labeled with Cy7, and both subcutaneous and orthotopic breast tumor models were established in BALB/c mice. Whole‐body fluorescence imaging and ex vivo biodistribution analyses were then performed at different time points.

To define the tissue distribution pattern of the nanoparticles, a subset of mice bearing subcutaneous tumors was euthanized at 24 h after administration, followed by ex vivo fluorescence imaging of tumors and major organs. As shown in Figure 6b, fluorescence signals from Cy7‐HSMIN‐LNP were predominantly detected in the tumor and liver, whereas only weak or nearly undetectable signals were observed in other major organs, including the heart, spleen, lung, and kidney. These findings indicate that HSMIN‐LNP possesses favorable tumor‐accumulation capability in vivo, while also undergoing a certain degree of hepatic uptake, suggesting potential involvement of liver‐associated clearance pathways in its metabolic disposition. It should be noted that, although HTIM modification was designed to enhance tumor‐cell recognition and targeted delivery, it did not completely eliminate the liver‐associated uptake commonly observed for intravenously administered LNPs. Such hepatic accumulation may be related to plasma protein adsorption, ApoE‐mediated hepatocyte uptake, and clearance by liver‐resident phagocytic cells, reflecting the intrinsic liver tropism of LNP‐based delivery systems [40]. Further optimization of LNP formulations may help improve tumor‐selective delivery in future studies.

Meanwhile, in vivo fluorescence imaging showed that, following tail‐vein injection of Cy7‐HSMIN‐LNP into mice bearing subcutaneous breast tumors, the nanoparticles rapidly reached the tumor site and exhibited evident fluorescence enrichment within the tumor region at early time points after administration. With increasing time, the fluorescence signal at the tumor site progressively intensified, reached peak accumulation at approximately 4–8 h post‐injection, and then gradually declined, becoming nearly undetectable by 72 h (Figure 6c). These findings indicate that HSMIN‐LNP possesses favorable tumor‐targeting capability in vivo and can be effectively cleared from the body within a relatively short time frame.

We then validated the in vivo distribution profile of Cy7‐HSMIN‐LNP in an orthotopic breast tumor model. Consistent with the subcutaneous model, ex vivo fluorescence imaging at 24 h likewise confirmed that the nanoparticles were predominantly distributed in the tumor and liver, whereas fluorescence signals in other major organs remained weak (Figure 6e). Whole‐body imaging further showed that Cy7‐HSMIN‐LNP effectively accumulated at the orthotopic tumor site, reached peak enrichment at approximately 4 h after administration, and was then gradually metabolized, with fluorescence signals becoming nearly undetectable by 72 h (Figure 6f). Together, these results demonstrate that HSMIN‐LNP exhibits consistent and stable tumor‐targeting distribution characteristics across different breast cancer models.

In addition, feces and urine were collected at each time point to evaluate the excretory behavior of HSMIN‐LNP. Detectable fluorescence signals were observed in both fecal and urinary samples as early as the initial post‐administration time points, followed by a gradual decline over time, with signals becoming nearly undetectable by 72 h (Figure 6g). These findings indicate that HSMIN‐LNP can be efficiently metabolized and excreted in vivo without evident long‐term retention. The above results demonstrate that HSMIN‐LNP exhibits favorable tumor‐targeting accumulation and controllable in vivo metabolic behavior in both subcutaneous and orthotopic TNBC models, thereby providing an important basis for subsequent therapeutic evaluation in animal studies.

Subsequently, we employed the Luc‐LNP system to evaluate the ability of the synthesized HTIM‐conjugated LNPs to deliver plasmids and induce transgene expression in vivo. In vivo bioluminescence imaging showed that no obvious luminescent signal was detected in subcutaneous 4T1 tumor‐bearing mice before administration. After intravenous injection of Luc‐LNPs via the tail vein, a distinct luciferase bioluminescence signal appeared in the tumor region and remained relatively strong from day 2 to day 10. Thereafter, the signal gradually decreased, although detectable luminescence was still observed on day 16 (Figure S7). These results demonstrate that Luc‐LNPs can effectively deliver luciferase‐encoding plasmids and achieve sustained transgene expression in mice, further confirming the feasibility of the synthesized LNPs as an in vivo plasmid delivery platform and providing experimental support for subsequent therapeutic gene expression‐based antitumor studies.

### 2.6 Sonogenetic Therapy Suppresses Tumor Growth and Activates Antitumor Inflammatory Signaling In Vivo

**FIGURE 7:**
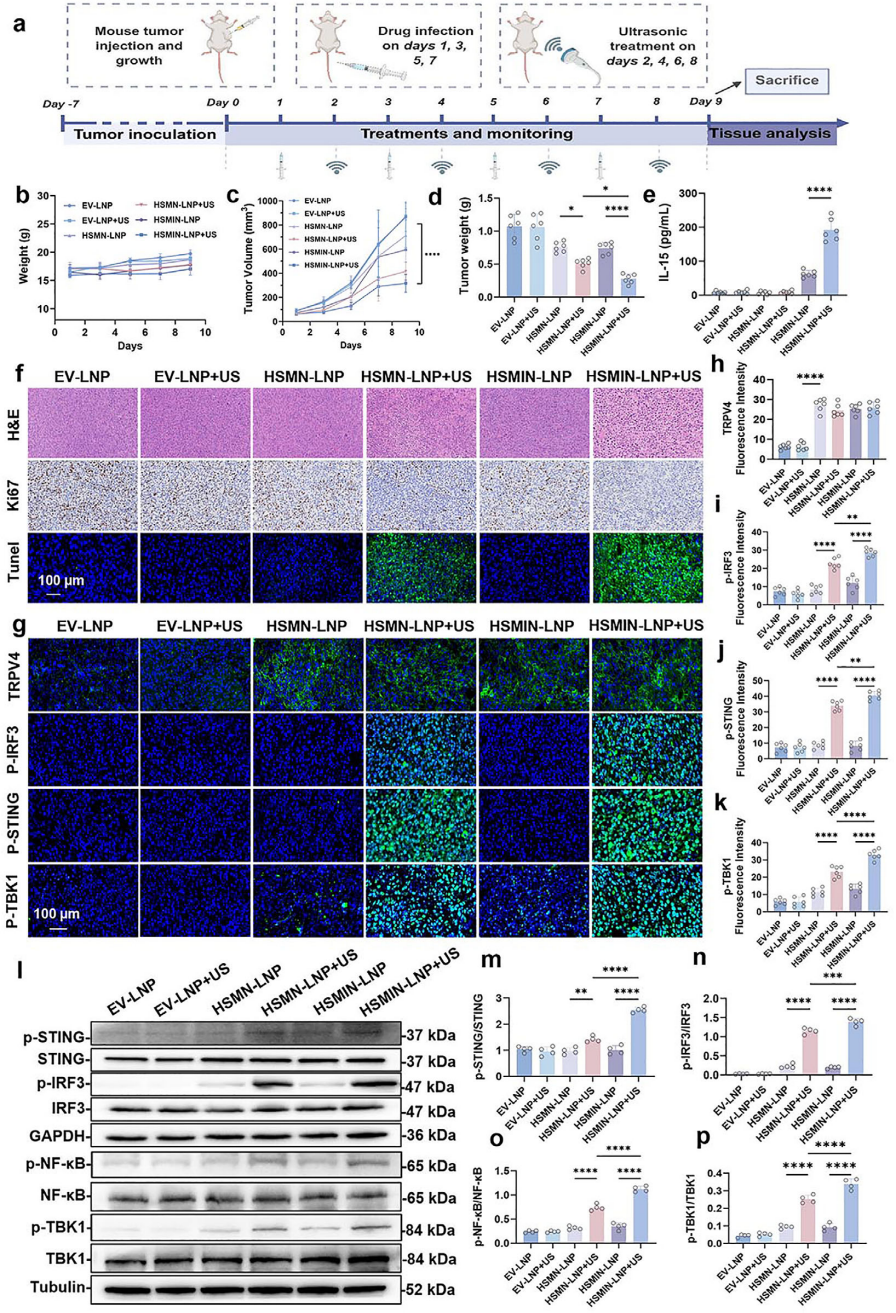
Ultrasound‐triggered HSMIN‐LNP treatment in 4T1 tumor‐bearing mice. (a) Schematic illustration of the in vivo experimental workflow. (b) Changes in body weight of mice during the treatment period. (c) Tumor growth curves over the treatment period for each group. (d) Quantification of average tumor weight for each treatment group at the study endpoint. (e) Quantification of IL‐15 levels in mouse serum measured by ELISA. (f) Representative images of tumor tissue stained with H&E (histological injury), Ki67 (cell proliferation), and TUNEL (apoptotic cells). Scale bar: 100 µm. (g) Representative immunofluorescence images of tumor sections showing TRPV4 expression and phosphorylation of IRF3, STING, and TBK1. Protein (green: FITC) and nuclei (blue: DAPI). Scale bar: 100 µm. (h–k) Quantification of fluorescence intensity for TRPV4, p‐IRF3, p‐STING, and p‐TBK1 corresponding to (g). (l) Western blot analysis of the phosphorylation levels of STING, IRF3, TBK1, and NF‐κB in tumor tissues under the indicated treatments. (m–p) Quantification of phosphorylated‐to‐total protein ratios for STING, IRF3, NF‐κB, and TBK1 corresponding to (l). Data are presented as mean ± SD (n = 6 or 4). The *p* values were calculated using one‐way ANOVA; ns: not significant, ^*^ *p* < 0.05, ^**^ *p* < 0.01, ^***^ *p* < 0.001, ^****^ *p* < 0.0001.

In the preceding experiments, we demonstrated that HSMIN‐LNP exhibits favorable tumor accumulation and suitable pharmacokinetic behavior in 4T1 tumor‐bearing mice. On this basis, we next evaluated the in vivo antitumor efficacy of this sonogenetic nanoplatform in a 4T1‐bearing BALB/c mouse model. As illustrated in Figure 7a, 4T1 cells were subcutaneously inoculated into the right axillary region of BALB/c mice. When tumor volumes reached approximately 100 mm^3^, tumor‐bearing mice were randomly assigned to six groups (n = 6) and administered EV‐LNP, HSMN‐LNP, or HSMIN‐LNP via tail‐vein injection. Based on the above biodistribution and expression profiles, the interval between nanoparticle administration and US stimulation was set at 24 h. Although Cy7‐HSMIN‐LNP exhibited peak tumor fluorescence at approximately 4–8 h post‐injection, tumor‐associated signals remained detectable at 24 h. In addition, Luc‐LNP imaging and the in vitro transfection optimization results indicated that plasmid‐mediated transgene expression persisted and became relatively stable around this time point. Therefore, US stimulation at 24 h post‐administration was selected to coordinate tumor accumulation with sufficient TRPV4 expression before acoustic activation. Considering that the 5 min in vitro US exposure effectively induced TRPV4/NFAT‐dependent gene activation and downstream cellular responses, the in vivo exposure duration was extended to 15 min to accommodate ultrasound attenuation, acoustic coupling variability, and the heterogeneous mechanical environment within solid tumors. Moreover, because LNP‐mediated plasmid expression is transient, the treatment was repeated for a total of four injection‐US cycles to recurrently reinforce TRPV4‐dependent Ca^2+^ signaling and NFAT‐mediated IL‐15 induction throughout the treatment course. During the 9‐day treatment and monitoring period, body weight and tumor volume were recorded every 2 days.

Body weight remained generally stable across all groups throughout the treatment period, with no evident loss observed (Figure 7b), indicating that the treatment regimen was well tolerated under the current dosing and stimulation conditions. In contrast, tumor growth curves diverged markedly among the treatment groups (Figure 7c). Compared with the EV‐LNP, EV‐LNP + US, and other single‐treatment groups, both the HSMN‐LNP + US and HSMIN‐LNP + US groups showed pronounced suppression of tumor growth, with the HSMIN‐LNP + US group exhibiting the most significant therapeutic effect. In this group, the mean tumor volume was reduced to approximately 42% of that in the control group. Endpoint tumor weight analysis further confirmed this trend, with the mean tumor weight in the HSMIN‐LNP + US group reduced to approximately 26% of that in the control group (Figure 7d). Together, these results demonstrate that this therapeutic strategy exerts potent antitumor activity in vivo.

Among the in vivo treatment groups, HSMIN‐LNP + US showed the most pronounced tumor growth inhibition and outperformed HSMN‐LNP + US. To investigate the underlying mechanism, and given that our in vitro studies had already demonstrated that HSMIN‐LNP + US markedly upregulated TRPV4 expression and induced robust IL‐15 secretion, we hypothesized that its superior in vivo efficacy might be closely associated with IL‐15‐mediated antitumor immune activation. On this basis, we first examined TRPV4 expression in tumor tissues. Western blot analysis showed that TRPV4 protein levels were significantly elevated in the HSMN‐LNP, HSMN‐LNP + US, HSMIN‐LNP, and HSMIN‐LNP + US groups following delivery of the pTrpv4 plasmid, and immunofluorescence analysis revealed a consistent trend (Figure S8a,b and Figure 7g). Furthermore, ELISA demonstrated that serum IL‐15 levels were markedly increased in the HSMIN‐LNP + US group, reaching approximately threefold higher than those in the corresponding non‐US group (Figure 7e). Together, these results indicate that LNP‐mediated delivery enables efficient TRPV4 overexpression in vivo, while US stimulation further triggers its functional activation.

Tumor histopathology was further examined to define the pathological basis underlying the observed antitumor effects. Hematoxylin and eosin (H&E) staining revealed marked structural disruption in tumor sections from the HSMN‐LNP + US and HSMIN‐LNP + US groups, characterized by disorganized cellular architecture, loss of tissue integrity, and focal necrosis‐like changes, with the most severe damage observed in the HSMIN‐LNP + US group. Consistent with this, immunohistochemical staining for Ki67, a marker of cell proliferation, showed that the HSMIN‐LNP + US group exhibited the lowest proportion of Ki67‐positive cells, indicating the most pronounced suppression of tumor proliferative activity. Likewise, terminal deoxynucleotidyl transferase dUTP nick end labeling (TUNEL) staining demonstrated a marked increase in tumor cell apoptosis in the HSMN‐LNP + US and HSMIN‐LNP + US groups, with the strongest green fluorescence signal detected in the HSMIN‐LNP + US group (Figure 7f). Together, these findings further demonstrate that HSMIN‐LNP combined with US more effectively suppresses tumor cell proliferation and promotes tumor cell apoptosis.

In the in vitro studies, we demonstrated that TRPV4 overexpression combined with US‐triggered Ca^2+^ overload induces mitochondrial depolarization, morphological disruption, and mtDNA release, thereby activating the cGAS‐STING pathway and eliciting downstream inflammatory and interferon‐related transcriptional programs. Based on this mechanism, we next assessed the activation status of the cGAS‐STING signaling axis in tumor tissues in vivo. Immunofluorescence showed that p‐IRF3, p‐STING, and p‐TBK1 signals were strongest in the HSMIN‐LNP + US group, exceeding those in the control and all single‐treatment groups. Although HSMN‐LNP + US also induced clear pathway activation, the signal intensity remained lower than that in the HSMIN‐LNP + US group (Figure 7g–k). Western blot analysis of tumor tissues further confirmed these findings (Figure 7l–p). Collectively, these results indicate that US‐triggered Ca^2+^ overload induces mitochondrial damage sufficient to activate the cGAS‐STING pathway in vivo, whereas enhanced IL‐15 secretion may further potentiate this signaling output, thereby cooperatively contributing to a stronger antitumor effect.

### 2.7 The Sonogenetic Nanoplatform Activates Antitumor Immune Responses In Vivo

**FIGURE 8:**
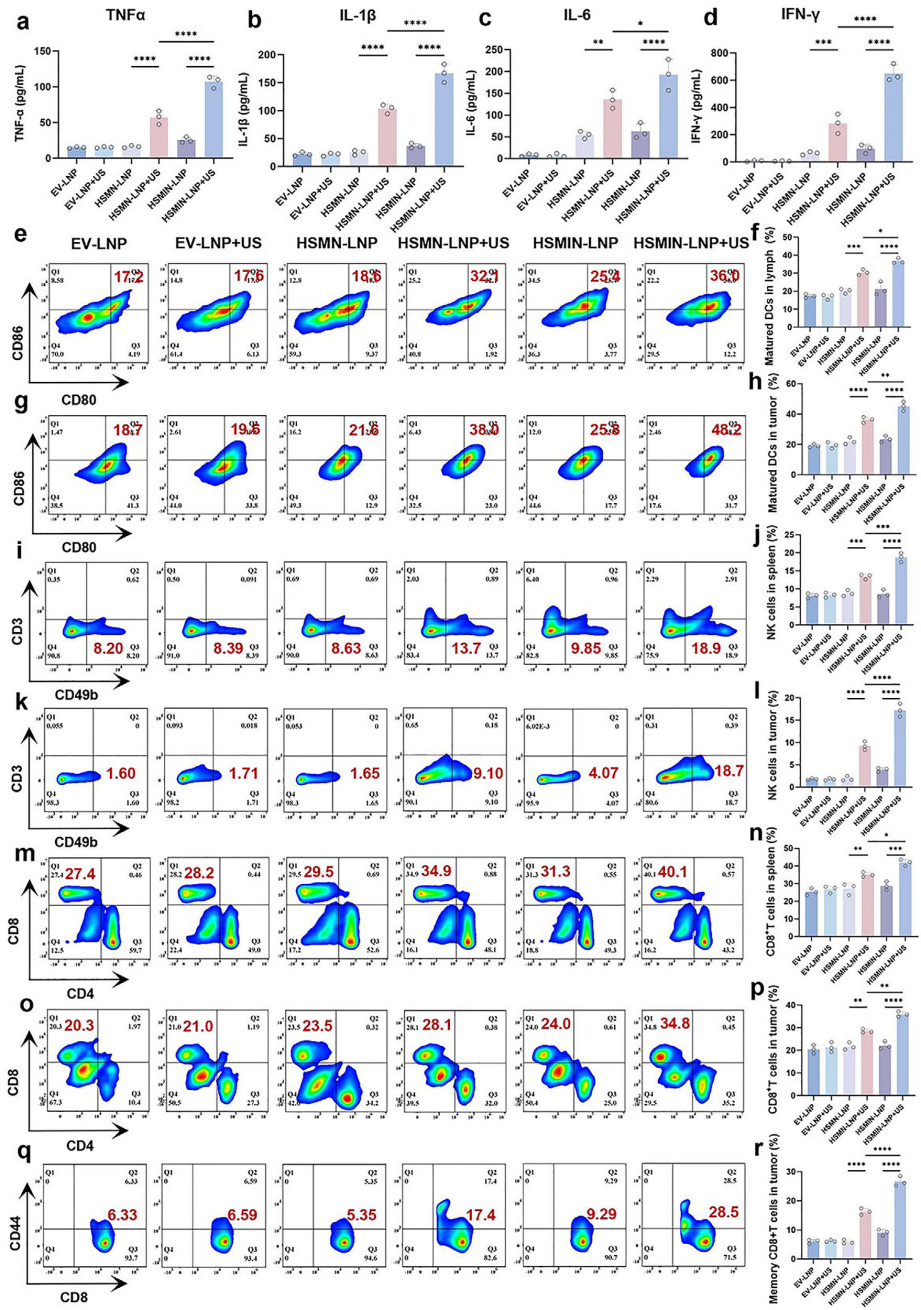
Evaluation of the in vivo antitumor immune activation effect of HSMIN‐LNP. (a–d) Serum levels of TNFα, IL‐1β, IL‐6, and IFN‐γ in mice under the indicated treatments. (e, f) Representative flow cytometry plots and corresponding quantification of mature DCs in lymphoid tissues under the indicated treatments. (g, h) Representative flow cytometry plots and corresponding quantification of mature DCs in tumor tissues under the indicated treatments. (i, j) Representative flow cytometry plots and corresponding quantification of NK cells in the spleen under the indicated treatments. (k, l) Representative flow cytometry plots and corresponding quantification of NK cells in tumor tissues under the indicated treatments. (m, n) Representative flow cytometry plots and corresponding quantification of CD8^+^ T cells in the spleen under the indicated treatments. (o, p) Representative flow cytometry plots and corresponding quantification of CD8^+^ T cells in tumor tissues under the indicated treatments. (q, r) Representative flow cytometry plots and corresponding quantification of memory CD8^+^ T cells in tumor tissues under the indicated treatments. Data are presented as mean ± SD (n = 3). The *p* values were calculated using one‐way ANOVA; ns: not significant, ^*^ *p* < 0.05, ^**^ *p* < 0.01, ^***^ *p* < 0.001, ^****^ *p* < 0.0001.

Based on the foregoing findings, we established that HSMIN‐LNP + US markedly activated the cGAS‐STING signaling pathway in tumor tissues. To determine whether this molecular activation could be translated into measurable in vivo immune outputs, we first examined changes in inflammatory and immune‐associated cytokines in mouse serum. ELISA analysis showed that, compared with the EV‐LNP, EV‐LNP + US, and other single‐treatment groups, serum levels of TNF‐α, IL‐1β, and IL‐6 were all significantly elevated in the HSMN‐LNP + US and HSMIN‐LNP + US groups, with the most pronounced increase observed in the HSMIN‐LNP + US group. This trend was consistent with the previously observed changes in phosphorylation levels of cGAS‐STING pathway‐associated proteins (Figure 8a–c). Meanwhile, serum IFN‐γ was also markedly increased in the HSMIN‐LNP + US group, reaching approximately 650 pg/mL, which was substantially higher than that in all other groups (Figure 8d).

Notably, in the preceding section, we proposed that the superior in vivo therapeutic efficacy of HSMIN‐LNP + US might be associated with IL‐15‐mediated antitumor immune activation, and accordingly demonstrated a significant increase in serum IL‐15 levels. Combined with the concomitant upregulation of multiple inflammatory cytokines and interferons observed here, these findings further suggest that this sonogenetic nanoplatform not only induces local activation of innate immune signaling, but may also enhance antitumor immune activation by promoting immune‐cell recruitment, maturation, and effector expansion.

To validate this hypothesis, we performed flow cytometric analysis of key immune cell populations in the spleen, lymphoid tissues, and tumor tissues. At the level of innate immune activation, the proportion of mature antigen‐presenting dendritic cells (DCs; CD11c CD80 CD86) in lymphoid tissues increased to approximately 36.0% in the HSMIN‐LNP + US group, significantly higher than that in the HSMN‐LNP + US group and all single‐treatment groups (Figure 8e,f). Within tumor tissues, the proportion of mature DCs further increased to approximately 48.2%, again markedly exceeding that in the HSMN‐LNP + US group (Figure 8g,h). In parallel, the proportion of NK cells (CD45 CD49b) in the spleen increased to approximately 18.9% in the HSMIN‐LNP + US group, compared with 13.7% in the HSMN‐LNP + US group (Figure 8i,j). Tumor‐infiltrating NK cells showed a similar trend, increasing to approximately 18.7% vs. 9.10% in the HSMN‐LNP + US group (Figure 8k,l). Together, these findings indicate that the sonogenetic nanoplatform robustly potentiates innate antitumor immunity by enhancing DC maturation, antigen presentation, and NK cell expansion and infiltration, thereby creating favorable conditions for the development of adaptive immune responses.

We further observed a marked enhancement of adaptive antitumor immunity. In the HSMIN‐LNP + US group, the proportion of CD8^+^ T cells (CD45 CD3 CD8) in the spleen increased to approximately 40.1%, clearly exceeding that in the HSMN‐LNP + US group and all other treatment groups (Figure 8m,n). Likewise, the proportion of tumor‐infiltrating CD8^+^ T cells increased to approximately 34.8%, compared with 28.1% in the HSMN‐LNP + US group (Figure 8o,p). These findings indicate that the sonogenetic nanoplatform not only promotes the expansion of cytotoxic T cells in peripheral immune organs, but also enhances their recruitment and infiltration into the tumor, thereby providing a cellular basis for direct tumor cell killing. More importantly, the proportion of memory CD8^+^ T cells (CD45 CD3 CD8 CD44) in tumor tissues was markedly increased to approximately 28.5% in the HSMIN‐LNP + US group, significantly higher than that in the HSMN‐LNP + US group (Figure 8q,r), indicating an enrichment of memory‐associated CD8^+^ T cell phenotypes following treatment. Collectively, these results indicate that the sonogenetic nanoplatform enhances systemic antitumor immune activation through the coordinated activation of innate and adaptive immunity. In doing so, it converts the elevated inflammatory cytokine signals described above into a multilayered, multicellular amplification of antitumor immunity in vivo.

Alongside evaluation of in vivo antitumor activity and immune modulation, we further performed a systematic assessment of biosafety. H&E staining of major organs revealed preserved histological architecture in the heart, liver, spleen, lung, and kidney across all treatment groups, with no evident tissue damage or inflammatory abnormalities (Figure S9a). Histological and immunofluorescence analyses revealed no significant changes in collagen deposition or fibrosis‐related marker expression following HSMIN‐LNP+US treatment, suggesting no detectable cardiac damage (Figure S9b). Serum biochemistry further showed no significant differences among groups in alanine aminotransferase (ALT) and aspartate aminotransferase (AST), reflecting hepatic function, or blood urea nitrogen (BUN) and creatinine (CRE), reflecting renal function (Figure S10a–d). Consistent with these findings, routine hematological analysis showed that white blood cells, lymphocytes, monocytes, granulocytes, red blood cells, hemoglobin, and platelets all remained within normal physiological ranges, without obvious treatment‐associated fluctuations (Figure S11a–h). Together, these data indicate that HSMIN‐LNP is well tolerated in vivo under the current dosing and US regimen and does not elicit detectable systemic toxicity.

Overall, this platform demonstrates promising translational potential by integrating tumor‐targeted delivery with therapeutic modulation of the tumor microenvironment, providing a feasible strategy for improving antitumor treatment efficacy. Nevertheless, the present study still has several limitations. In particular, although the current results support the short‐term therapeutic activity and mechanistic feasibility of this system, long‐term endpoint studies evaluating durable tumor suppression, recurrence control, survival benefit, and potential systemic toxicity remain necessary. Future work should further optimize the formulation parameters, dosing regimen, biodistribution profile, and safety window, as well as validate the therapeutic performance in more clinically relevant tumor models. These efforts will help clarify the robustness and translational applicability of this platform for future antitumor therapy.

## 3 Conclusion

In summary, we developed HSMIN‐LNP, a HER3‐targeted sonogenetic mechano‐immunomodulatory nanoplatform, that integrates targeted receptor inhibition, US‐gated mechanotransduction, and immune activation for the treatment of TNBC. Built on the AI‐designed HER3‐targeting miniprotein HTIM, this platform effectively suppressed HER3 and its migration‐associated downstream signaling pathways. Using LNPs as the delivery vehicle, HSMIN‐LNP co‐encapsulated a TRPV4 expression plasmid and an NFAT‐responsive IL‐15 expression construct. Meanwhile, HTIM was conjugated to the nanoparticle surface through a TK linker, enabling its US‐responsive release and thereby establishing a dual‐regulatory system that combines extracellular receptor blockade with intracellular gene activation. Mechanistically, US‐triggered TRPV4 activation drives Ca^2+^ overload, which not only promotes NFAT‐dependent IL‐15 induction but also elicits oxidative stress, mitochondrial dysfunction, apoptosis, and cGAS‐STING‐mediated inflammatory signaling, thereby linking direct tumor killing to antitumor immune activation. In vivo, HSMIN‐LNP showed favorable tumor accumulation, biosafety, and therapeutic efficacy, while enhancing DC maturation, NK cell and CD8^+^ T cell activation, with an increased proportion of memory CD8^+^ T cells. Overall, this study presents a US‐triggered synergistic therapeutic strategy that integrates mechanosensitive channel regulation, targeted blockade, and antitumor immune activation within a single platform, and provides a promising framework for the precision treatment of TNBC and potentially other solid tumors.

## 4 Materials and Methods

### 4.1 Materials

DSPE‐PEG_2000_‐TK‐Mal and DSPE‐PEG_2000_‐Cy7 were custom synthesized by Tanshtech. Cholesterol (HY‐N0322), DSPE‐PEG_2000_ (HY‐142979), DOTAP (HY‐112754A), and SM‐102 (HY‐134541) were purchased from MedChemExpress. Recombinant human HER3/ERBB3 protein (10201‐H08H) was obtained from Sino Biological. SSA biosensors (18‐5057) were purchased from Sartorius. Roswell Park Memorial Institute (RPMI) 1640 medium (C1875500BT), Trypsin‐EDTA (25200072), Opti‐MEM reduced‐serum medium (31985062), and TRIzol reagent (15596018CN) were purchased from Thermo Fisher Scientific. Fetal bovine serum (FBS, FSP500) was purchased from ExCell Bio. 4T1 complete medium (CM‐0007) was purchased from Procell. Penicillin‐streptomycin (PS; C100C5) and phosphate‐buffered saline (PBS; C500C1) were purchased from New Cell & Molecular Biotech. ElaBoX Mouse IL‐15 ELISA Kit (SEKM‐0015), BCA protein assay kit (PC0020), Hoechst 33342 (C0031), reactive oxygen species assay kit (CA1410), Annexin V‐FITC/PI apoptosis detection kit (CA1020), Calcein‐AM/PI live/dead cell double‐staining kit (CA1630), mitochondrial membrane potential kit (CA1310), MitoTracker Red CMXRos (M9940), MitoSOX Red (IYT51679), DAPI solution (C0060), high‐efficiency RIPA lysis buffer (R0010), 5% bovine serum albumin (BSA; SW3015), D‐Hanks (H1045), and HBSS (H1025) were purchased from Solarbio. Cal‐520 AM (21130) was purchased from AAT Bioquest. dsDNA HS Assay Kit (12640ES76), Hifair AdvanceFast first Strand cDNA Synthesis Kit (11150ES60), and Hieff UNICON Advanced qPCR SYBR Master Mix (11185ES08) were purchased from Yeasen Biotechnology. The following primary antibodies were purchased from Cell Signaling Technology (CST): mTOR antibody (2972), phospho‐mTOR (Ser2448) antibody (2971), HER3 antibody (12708), phospho‐HER3 (Tyr1289) antibody (4791), Akt antibody (9272), phospho‐Akt (Ser473) antibody (9271), ERK1/2 antibody (4695), phospho‐ERK1/2 (Thr202/Tyr204) antibody (9101), STING antibody (13647), phospho‐STING (Ser365) antibody (72971/51865), TBK1 antibody (3504), phospho‐TBK1 (Ser172) antibody (5483), phospho‐NF‐κB p65 (Ser536) antibody (3033), and phospho‐IRF3 (Ser396) antibody (29047). The following antibodies were purchased from Abcam: anti‐TRPV4 antibody (ab191580), anti‐E‐cadherin antibody (ab231303), anti‐N‐cadherin antibody (ab18203), anti‐MMP9 antibody (ab228402), anti‐NF‐κB p65 antibody (ab32536), and anti‐IRF3 antibody (ab68481). β‐Tubulin monoclonal antibody (66240‐1‐Ig), GAPDH monoclonal antibody (60004‐1‐Ig), HRP‐conjugated goat anti‐mouse IgG (H+L) (SA00001‐1), and HRP‐conjugated goat anti‐rabbit IgG (H+L) (SA00001‐2) were purchased from Proteintech. All antibodies used for flow cytometry are listed in Table S5.

### 4.2 Computational Design of Miniproteins

The inactive HER3 crystal structure (PDB ID: 1M6B) and active‐state cryo‐EM structure (PDB ID: 7MN5) were used as structural templates for computational miniprotein design. As domain I of HER3 (residues S25‐T206) is largely conserved between the inactive and active conformations, this region was selected as the target surface for miniprotein design. Solvent‐exposed residues within domain I, including L33, L36, Y86, L88, M91, Y111, F115, Y148, E150, and K177, were defined as hotspot residues to guide backbone generation. RFdiffusion was then used to generate miniprotein backbones with a target length of 60–100 amino acids through a 50‐step diffusion process, using a noise scale of 0.5. Approximately 1000 candidate protein backbones were generated and subsequently subjected to sequence design using ProteinMPNN. For each backbone, about 100 sequences were designed in the structural context of HER3 domain I using random seeds, the v_48_020 checkpoint, and a sampling temperature of 0.1. In total, approximately 100 000 designed sequences were evaluated with AlphaFold3, and the candidates were ranked according to the predicted interface TM‐score (ipTM), which was used as a confidence metric for HER3 binding. Finally, five designs with distinct structural folds were selected for experimental characterization based on structural diversity analysis using a pairwise RMSD cutoff of 2.5 Å.

### 4.3 Protein Purification

The genes encoding the designed miniproteins were synthesized and cloned into the pET28a vector by TsingKe Biotechnology (Beijing, China). Recombinant plasmids were transformed into E. coli BL21 (DE3) cells by heat shock. The cells were cultured in Luria‐Bertani (LB) medium containing kanamycin at 37°C to an OD_600_ of approximately 0.6, and protein expression was induced with 0.5–1.0 mm IPTG at 16°C for 18 h. After harvesting by centrifugation at 4000 rpm, the cell pellets were resuspended in lysis buffer (20 mm Tris‐HCl, pH 7.5, 200 mm NaCl) and disrupted by sonication. The lysate was clarified by centrifugation at 16 000 rpm for 40 min, and the supernatant was loaded onto Ni‐NTA resin. The resin was washed with lysis buffer containing 30 mm imidazole, and the bound proteins were eluted with 300 mm imidazole in the same buffer. The eluates were concentrated by ultrafiltration at 2900 rpm and further purified by size‐exclusion chromatography using a Superdex 75 Increase 10/300 GL column (Cytiva, Shanghai, China) equilibrated with PBS (150 mm NaCl, 15 mm Na_2_HPO_4_, 5 mm NaH_2_PO_4_, pH 7.4). Purified proteins were quantified by BCA assay, flash‐frozen in liquid nitrogen, and stored at ‐80°C for subsequent use.

### 4.4 Bio‐Layer Interferometry (BLI) Binding Assay

Biotinylated proteins were immobilized onto super streptavidin (SSA) biosensors to a loading response of 3 nm. After immobilization, the biosensors were blocked in kinetics buffer consisting of PBS supplemented with 0.02% (v/v) Tween 20 and 1 mg/mL BSA, followed by incubation with test compounds at different concentrations and subsequent dissociation in the same kinetics buffer. Each step was carried out for 300 s. Real‐time binding signals were recorded using an Octet HTX instrument and analyzed with Octet HT software v10.0.

### 4.5 Cell Lines and Animals

4T1 cells were purchased from Procell Life Science & Technology Co., Ltd. (Wuhan, China) and cultured in a 2:1 mixture of RPMI 1640 medium supplemented with 10% (v/v) FBS and 1% (v/v) PS and 4T1 Cell Complete Medium at 37°C in 5% CO_2_. Female BALB/c mice (6–8 weeks old) were obtained from Beijing Vital River Laboratory Animal Technology Co., Ltd. (Beijing, P. R. China) and housed under specific pathogen‐free conditions at 22°C–26°C with ad libitum access to standard chow and filtered tap water. All animal procedures were performed in accordance with national guidelines for laboratory animal welfare and ethics (approval no. ZXHK‐DWLL‐2026‐0054).

### 4.6 Plasmid Construction

Plasmid vectors *pECMV‐MCS‐FLAG*, *pCMV‐GFP*, and *pECMV‐Trpv4‐m‐FLAG* were obtained from Miaoling Biotechnology Co., Ltd. (Wuhan, China). The *pGL4.50[luc2/CMV/Hygro]* plasmid was purchased from Promega Corporation (Madison, WI, USA). Among these plasmids, *pECMV‐MCS‐FLAG* served as the empty vector for EV‐LNP formulation, *pCMV‐GFP* was used for transfection optimization, *pECMV‐Trpv4‐m‐FLAG* was used for TRPV4 overexpression in cells and in mice, and *pGL4.50[luc2/CMV/Hygro]* was used to assess transgene expression in mice, generating luciferase‐expressing LNPs (Luc‐LNPs). A human‐mouse codon‐co‐optimized IL‐15 coding sequence was synthesized by Genewiz (Suzhou, China) and inserted into *pGL4.30[luc2P‐NFAT‐RE‐Hygro]* (Promega, USA) to generate *pGL4.30[NFAT‐RE‐IL15‐Hygro]*. To enable evaluation of ultrasound‐responsive expression, mCherry was introduced downstream of IL‐15 by Gibson assembly, yielding *pGL4.30[NFAT‐RE‐IL15‐P2A‐mCherry‐Hygro]*. Detailed sequence information is provided in Tables S1 and S2.

### 4.7 Ultrasound Equipment and Parameters

In this study, LIPUS was used for cellular and in vivo stimulation. US signals were generated by an arbitrary waveform generator, amplified by a power amplifier, and then delivered through a planar piezoelectric transducer. Specifically, a Keysight 33600A Series Trueform waveform generator (50 Ω output system) was used to generate a 1 MHz sinusoidal carrier signal with pulse gating, and a Mini‐Circuits LZY‐22^+^ power amplifier (50 Ω system; typical gain, ∼43 dB; operating frequency range, 0.1–200 MHz) was used to amplify the low‐amplitude electrical signal to a level sufficient to drive the transducer. A custom planar US transducer (CSS‐TC‐1000K‐D20; center frequency, 1 MHz; nominal output impedance, 50 Ω) was used as the acoustic output device. To ensure consistency of the delivered acoustic dose across different experimental batches and well positions, the acoustic output of the transducer was calibrated under different peak‐to‐peak driving voltages (Vpp). Table S3 summarizes the relationship between Vpp and calibrated acoustic power under continuous‐wave conditions. The continuous‐wave calibrated power (P_CW_) was further converted to time‐averaged acoustic power (P_TA_) according to the duty cycle (DC). All experiments were performed within the safe operating range of the transducer, with the driving voltage maintained below the upper limit (≤120 Vpp). In addition, the output waveform was verified before and after each experiment to exclude possible effects of gating distortion or output drift on dose consistency.

For in vitro experiments, US stimulation was applied in 12‐well plates. Before stimulation, the transducer was positioned directly above the target well using a fixed clamp. Each well was filled with culture medium, and bubble entrapment was minimized to reduce errors caused by acoustic reflection and local field inhomogeneity. The LIPUS parameters were set as follows: center frequency, 1 MHz; pulse repetition frequency (PRF), 1 kHz; DC, 20%; and stimulation duration, 5 min per treatment. For in vivo experiments, the transducer was placed directly over the tumor region of mice, and US coupling gel was evenly applied at the interface between the transducer surface and the skin to ensure stable acoustic coupling. The in vivo LIPUS settings, including frequency, PRF, and DC, were identical to those used in vitro, whereas the stimulation duration was extended to 15 min.

### 4.8 Preparation and Characterization of HSMIN‐LNP

HSMIN‐LNP was prepared through the self‐assembly of a lipid phase and an aqueous phase. The lipid phase was dissolved in anhydrous ethanol and consisted of four components: SM‐102, cholesterol, DOTAP, and DSPE‐PEG_2000_, at a molar ratio of 50:38.5:10:1.5. The aqueous phase consisted of sodium citrate buffer (pH∼4.0), in which plasmids were dissolved. The lipid and aqueous phases were rapidly mixed at a volume ratio of 3:1, with a total lipid‐to‐plasmid mass ratio of 40:1 (w/w). The mixture was then incubated at room temperature for 15 min to allow nanoparticle formation. To obtain the DSPE‐PEG_2000_‐TK‐HTIM component, purified HTIM was conjugated to DSPE‐PEG_2000_‐TK‐Mal at a molar ratio of 1.2:1 in PBS (pH∼6.5–7.5). The reaction was carried out under argon protection in the presence of 0.1% triethylamine as a catalyst and stirred at room temperature for 24 h. After completion of the reaction, unreacted reagents were removed by dialysis. The resulting product was then lyophilized, dissolved in D_2_O, and analyzed by ^1^H NMR to confirm successful conjugation. Finally, the synthesized DSPE‐PEG_2000_‐TK‐HTIM was incorporated onto the surface of LNPs using a post‐insertion method.

The hydrodynamic diameter distribution and zeta potential of the synthesized LNPs were characterized using a dynamic light scattering analyzer (Zetasizer, Malvern, UK). Morphological analysis was performed by TEM (TECNAI G2 F20, Philips, Netherlands). Colloidal stability was evaluated by monitoring the time‐dependent changes in particle size in different solvent systems. The structural integrity of LNPs and the stability of the encapsulated plasmids were assessed by gel electrophoresis using a Bio‐Rad system (Hercules, CA, USA) in 1× Tris‐EDTA (TE) buffer. Plasmid encapsulation efficiency was indirectly determined using a dsDNA HS Assay Kit. Briefly, LNPs were dispersed separately in TE buffer and 2% Triton X‐100‐containing TE buffer for fluorescence measurement. The fluorescence signal measured in TE buffer represented the amount of free plasmid not encapsulated by LNPs, whereas the signal measured in 2% Triton X‐100 TE buffer represented the total plasmid content. Based on the difference between these two measurements, the encapsulation efficiency of plasmids was calculated.

### 4.9 Transwell and Wound‐Healing Assays

For Transwell assays, 4T1 cells (5 × 10^4^ cells in 200 µL per well) were seeded into the upper chamber of 12‐well Transwell inserts, while the lower chamber contained complete medium supplemented with 20% FBS and 10 µg/mL HTIM. After 24 h at 37°C, migrated cells on the lower membrane surface were washed with PBS, fixed with paraformaldehyde, stained with crystal violet, and counted in multiple random microscopic fields. For wound‐healing assays, 4T1 cells were seeded into 12‐well plates at 1 × 10^5^ cells/mL. After 24 h, a linear scratch was generated using a sterile pipette tip. Cells were washed three times with PBS and then incubated in serum‐free medium with or without 10 µg/mL HTIM for 24 h at 37°C. Wound closure was imaged under a microscope and quantified by measuring the migration distance. Wound healing rate (%) = (L_0_‐L_1_)/L_0×100_%.

### 4.10 Transfection

4T1 cells were seeded into 12‐well plates at a density of 2 × 10^5^ cells/mL. In the EV‐LNP and EV‐LNP + US groups, cells were transfected with 4 µg of *pECMV‐MCS‐FLAG* per well. In the HSMN‐LNP and HSMN‐LNP + US groups, cells were transfected with 2 µg of *pECMV‐MCS‐FLAG* and 2 µg of *pECMV‐Trpv4‐m‐FLAG* per well. In the HSMIN‐LNP and HSMIN‐LNP + US groups, cells were transfected with 2 µg of *pECMV‐Trpv4‐m‐FLAG* and 2 µg of *pGL4.30[NFAT‐RE‐IL15‐Hygro]* per well. Notably, the LNPs used in the EV‐LNP and EV‐LNP + US groups were not modified with HTIM, whereas LNPs in all other groups were surface‐functionalized with HTIM by default. After incubation under these conditions at 37°C for 3 h, the medium was replaced with complete medium containing 10% FBS. Cells were then further incubated for 24 h before being collected for subsequent analyses.

### 4.11 Immunofluorescence Staining of TRPV4

For TRPV4 immunofluorescence staining, 4T1 cells were seeded into 12‐well plates at 2 × 10^5^ cells/mL. When confluence reached approximately 80%, cells were incubated with EV‐LNP, HSMN‐LNP, or HSMIN‐LNP for 3 h, cultured for an additional 9 h at 37°C, and then exposed to US in the EV‐LNP + US, HSMN‐LNP + US, and HSMIN‐LNP + US groups, followed by 12 h of further incubation. Cells were fixed with 4% paraformaldehyde for 15 min, blocked with 1% BSA for 20 min, and incubated with an anti‐TRPV4 primary antibody overnight at 4°C. After washing with TBST, cells were incubated with FITC‐labeled goat anti‐mouse IgG (H+L) for 30 min at room temperature in the dark, counterstained with DAPI, and imaged using a confocal laser scanning microscope (CLSM, Leica Microsystems STELLARIS DMi8). Fluorescence images were analyzed using ImageJ.

### 4.12 Intracellular Free Ca^2+^ Detection

Intracellular Ca^2+^ was monitored using Cal‐520 AM (Ex, 490 nm; Em, 525 nm). 4T1 cells were incubated with EV‐LNP, HSMN‐LNP, or HSMIN‐LNP for 3 h and cultured for an additional 21 h at 37°C. Before imaging, cells were washed with Hanks’ balanced salt solution without calcium and magnesium (D‐Hanks), loaded with Cal‐520 AM for 30 min at 37°C in the dark, and counterstained with Hoechst 33342 for 10 min. Fluorescence images were first collected from a fixed field under CLSM before US stimulation. The same field was then stimulated by US without changing plate position, and images were acquired immediately afterward. The extracellular solution was subsequently replaced with HBSS, and the same procedure was repeated to compare US‐induced intracellular Ca^2+^ changes under Ca^2+^‐free and Ca^2+^‐containing conditions.

### 4.13 Determination of Intracellular ROS Levels

For intracellular ROS detection, 4T1 cells were seeded into 12‐well plates at 2 × 10^5^ cells/mL and incubated with EV‐LNP, HSMN‐LNP, or HSMIN‐LNP for 3 h when confluence reached approximately 80%. After 9 h of further culture at 37°C, the EV‐LNP + US, HSMN‐LNP + US, and HSMIN‐LNP + US groups were exposed to US, followed by an additional 12 h of incubation. Cells were then washed with PBS and stained with 0.02% DCFH‐DA for 20 min at 37°C in the dark. After washing, cells were counterstained with Hoechst 33342 and either imaged by CLSM or harvested by trypsinization for flow cytometric analysis (CytoFLEX LX, Beckman Coulter) of DCF fluorescence to quantify intracellular ROS.

### 4.14 Live/Dead Cell Staining Assay

4T1 cells were seeded into 12‐well plates, and cell viability was assessed using a Calcein‐AM/PI live/dead cell double‐staining kit. Cell treatment conditions were the same as described above. At the end of treatment, the culture medium was removed, and cells were washed twice with PBS. Cells were then incubated with a staining solution containing 2 µm Calcein‐AM and 5 µm propidium iodide (PI) at 37°C for 30 min in the dark. After washing with PBS, fluorescence images were acquired by CLSM and analyzed using ImageJ.

### 4.15 Flow Cytometry Analysis of Apoptosis

4T1 cells were seeded into 12‐well plates, and apoptosis was evaluated using an Annexin V‐FITC/PI apoptosis detection kit. Cell treatment conditions were the same as described above. After treatment, the culture medium was removed and cells were washed twice with PBS. Cells were then incubated with 5 µL Annexin V‐FITC working solution at 37°C for 10 min in the dark. After incubation, cells were washed with 1× binding buffer, collected by trypsinization, and resuspended in the same buffer. Subsequently, 5 µL PI working solution was added to the cell suspension, and samples were analyzed by flow cytometry. Apoptosis levels were quantified using FlowJo software.

### 4.16 Analysis of Mitochondrial Membrane Potential (ΔΨm)

For mitochondrial membrane potential analysis, 4T1 cells cultured in 12‐well plates were treated as described above. At the end of treatment, the culture medium was removed, and cells were washed twice with PBS. Cells were then incubated with JC‐10 working solution at 37°C for 20 min in the dark. After incubation, cells were washed twice with the buffer provided in the JC‐10 kit, collected by trypsinization, and resuspended in the same buffer. Cell suspensions were then immediately analyzed by flow cytometry, and fluorescence intensity was quantified using FlowJo software to evaluate changes in mitochondrial membrane potential.

### 4.17 Detection of Mitochondrial Morphology Disruption

4T1 cells were seeded into 12‐well plates, and mitochondrial morphology was assessed using MitoTracker Red CMXRos. Cell treatment conditions were the same as described above. At the end of treatment, the culture medium was removed, and cells were washed twice with PBS. Cells were then incubated with 200 nm MitoTracker working solution at 37°C for 30 min in the dark. After incubation, cells were washed twice with PBS and counterstained with Hoechst 33342 for 10 min, followed by two additional washes. Fluorescence images were then acquired using a CLSM and analyzed with ImageJ in combination with the MiNA plugin.

### 4.18 Mitochondrial Superoxide Assay

4T1 cells were seeded into 12‐well plates, and mitochondrial oxidative stress was assessed using MitoSOX Red. Cell treatment conditions were the same as described above. At the end of treatment, the culture medium was removed, and cells were washed twice with PBS. Cells were then incubated with 1 µm MitoSOX working solution at 37°C for 20 min in the dark. After incubation, cells were washed twice with PBS and counterstained with Hoechst 33342 for 10 min, followed by two additional washes. Cells were then either directly imaged using a CLSM or collected after trypsinization, resuspended in PBS, and analyzed by flow cytometry to measure MitoSOX fluorescence intensity as an indicator of mitochondrial superoxide levels.

### 4.19 Western Blotting

Treated cells were collected and lysed in RIPA buffer supplemented with 1% protease inhibitor. For tumor tissue samples, freshly isolated mouse tumors were minced into small pieces, homogenized in an appropriate volume of high‐efficiency RIPA lysis buffer using a tissue homogenizer, and centrifuged to collect the supernatant for subsequent analysis. Protein concentrations were determined using the BCA assay. Protein samples were then mixed with 5× loading buffer and denatured at 99°C for 10 min in a metal bath. Equal amounts of protein were separated by SDS‐PAGE and transferred onto nitrocellulose (NC) membranes. Membranes were blocked in TBST containing 5% nonfat milk for 30 min at room temperature with gentle shaking, followed by incubation with the indicated primary antibodies overnight at 4°C. On the following day, membranes were washed three times with TBST and then incubated with horseradish peroxidase (HRP)‐conjugated secondary antibodies for 1 h at room temperature. After incubation, membranes were washed three additional times with TBST, and protein bands were visualized using an enhanced chemiluminescence (ECL) detection system. Band intensities were quantified by densitometric analysis using ImageJ.

### 4.20 Quantitative Real‐Time Polymerase Chain Reaction (qPCR)

Treated cells were collected, and total RNA was extracted using TRIzol reagent. RNA concentration and purity were determined by spectrophotometry. Total RNA was then reverse‐transcribed into complementary DNA (cDNA) using the Hifair AdvanceFast first Strand cDNA Synthesis Kit. qPCR was performed on a QuantStudio real‐time PCR system using Hieff UNICON Advanced qPCR SYBR Master Mix. β‐Actin was used as the internal reference gene, and relative gene expression levels were calculated using the 2^−ΔΔCt^ method. All primers used in this study were designed based on the PrimerBank database, and the corresponding sequences are listed in Table S4.

### 4.21 Enzyme‐Linked Immunosorbent Assay (ELISA)

For in vitro experiments, culture supernatants were collected after treatment and centrifuged to remove cellular debris. For in vivo experiments, whole‐blood samples were collected from mice and allowed to clot at room temperature, followed by centrifugation to obtain serum. The levels of target factors in cell culture supernatants or serum were then measured using commercial ELISA kits according to the manufacturer's instructions. After termination of the enzymatic reaction, absorbance was measured at 450 and 630 nm using a microplate reader. The absorbance at 630 nm was used as the background correction value and subtracted from the corresponding reading at 450 nm. Concentrations of the indicated factors were finally calculated based on the standard curves.

### 4.22 In Vivo Fluorescence Imaging in Mice

Female BALB/c mice (6–8 weeks old) were randomly divided into two groups (n = 6) for establishment of subcutaneous and orthotopic breast tumor models, respectively. For the subcutaneous breast tumor model, 4T1 cells were inoculated subcutaneously into the right axillary region of each mouse. For the orthotopic breast tumor model, 4T1 cells were inoculated into the mammary fat pad adjacent to the fourth nipple. When tumor volumes reached approximately 100 mm^3^, mice were intravenously injected via the tail vein with Cy7‐HSMIN‐LNP loaded with 25 µg plasmid per mouse. In each model, two mice receiving PBS by tail‐vein injection were included as controls. Fluorescence imaging was performed using an in vivo imaging system before administration and at 2, 4, 8, 24, 48, and 72 h after injection. At the same time points, fecal and urinary samples were collected and subjected to fluorescence imaging to evaluate the in vivo metabolism and excretion of the nanoparticles. In addition, a subset of mice was euthanized at 24 h post‐injection, and the tumor and major organs, including the heart, liver, spleen, lung, and kidney, were harvested for ex vivo fluorescence imaging to assess the biodistribution of Cy7‐HSMIN‐LNP in tumors and normal tissues.

Meanwhile, to further evaluate the ability of LNPs to mediate plasmid delivery and induce transgene expression in vivo, Luc‐LNPs loaded with the *pGL4.50[luc2/CMV/Hygro]* plasmid were administered to subcutaneous 4T1 tumor‐bearing BALB/c mice. Briefly, when the subcutaneous tumor volume reached approximately 100 mm^3^, mice were injected with Luc‐LNPs via the tail vein, and subjected to in vivo bioluminescence imaging on days 0, 2, 4, 6, 8, 10, 12, 14, and 16 after administration. Before imaging, mice were anesthetized with 3% isoflurane and intraperitoneally injected with 100 µl of D‐luciferin sodium salt solution (30 mg mL^−1^). The mice were then placed in the lateral position in an in vivo imaging system for whole‐body bioluminescence signal acquisition.

### 4.23 In Vivo Antitumor Efficacy Evaluation

4T1 cells were subcutaneously inoculated into the right axillary region of BALB/c mice. When tumor volumes reached approximately 100 mm^3^, tumor‐bearing mice were randomly assigned to six groups (n = 6) and intravenously administered EV‐LNP, HSMN‐LNP, or HSMIN‐LNP at a dose of 25 µg pDNA per mouse. At 24 h after each administration, the tumor region was subjected to US stimulation, and this treatment cycle was repeated for a total of four cycles. During the 9‐day treatment and monitoring period, body weight and tumor volume were recorded every 2 days. At the end of the experiment, mice were euthanized. Blood samples were collected for complete blood count analysis, serum biochemistry, and ELISA. Major organs, including the heart, liver, spleen, lung, and kidney, were harvested, fixed in 4% paraformaldehyde, embedded in paraffin, and subjected to H&E staining to evaluate in vivo safety. Tumors were excised and weighed, and then processed for subsequent analyses. A portion of the tumor tissue was fixed for H&E staining, TUNEL staining, and Ki67 immunohistochemical staining to assess histopathological changes. Another portion was homogenized for Western blot analysis. The remaining tumor tissue was prepared as frozen sections for immunofluorescence staining of TRPV4, p‐IRF3, p‐STING, and p‐TBK1.

### 4.24 Immune Cell Infiltration

4T1 cells were subcutaneously inoculated into the right axillary region of BALB/c mice. When tumor volumes reached approximately 100 mm^3^, tumor‐bearing mice were randomly assigned to six groups (n = 3), and subsequent treatments were performed as described above. At the end of the experiment, mice were euthanized, and tumors, spleens, and tumor‐draining lymph nodes were collected. Tissues were minced into small pieces and passed through 70 µm cell strainers to prepare single‐cell suspensions, followed by red blood cell lysis. To evaluate DC maturation in vivo, a portion of the tumor and lymph node samples was blocked with 5% BSA and then incubated with PE anti‐mouse CD11c, APC anti‐mouse CD80, and FITC anti‐mouse CD86 antibodies for 30 min at 4°C in the dark. After staining, cells were washed twice with PBS, resuspended in PBS, and subjected to flow cytometric analysis. To assess NK cell infiltration, a portion of the spleen and tumor samples was stained with FITC anti‐mouse CD3, Pacific Blue anti‐mouse CD45, and APC anti‐mouse CD49b antibodies, followed by flow cytometric analysis. To evaluate CD8^+^ T cell activation, a portion of the spleen and tumor samples was stained with PE/Cyanine7 anti‐mouse CD45, FITC anti‐mouse CD3, APC anti‐mouse CD4, and PE anti‐mouse CD8a antibodies, followed by flow cytometric analysis. To determine the proportion of memory CD8^+^ T cells, a portion of the tumor samples was stained with PE/Cyanine7 anti‐mouse CD45, FITC anti‐mouse CD3, PE anti‐mouse CD8a, and Pacific Blue anti‐mouse CD44 antibodies, followed by flow cytometric analysis. All antibodies used in the above assays were purchased from BioLegend.

### 4.25 Statistical Analysis

All data were analyzed using GraphPad Prism 10 software and are presented as mean ± SD. Statistical significance was determined by one‐way analysis of variance (ANOVA) followed by Tukey's multiple‐comparisons test. Significance levels were defined as follows: ^*^ *p* < 0.05, ^**^ *p* < 0.01, ^***^ *p* < 0.001, ^****^ *p* < 0.0001.

## Author Contributions

**Junya Song**: investigation, methodology, resources, experiments, data analysis, and Writing – original draft; **Yan Liu**: methodology and visualization. **Shifeng Zhao**: validation and analysis; **Cheng Zhu**: conceptualization, funding acquisition, and writing – review and editing. **Hongchang Guo**: conceptualization and supervision. **Jin Chang**: supervision, funding acquisition, and writing – review and editing. **Jun Kang**: conceptualization, supervision, and writing – review and editing.

## Conflicts of Interest

The authors declare no conflicts of interest.

## Supporting information




**Supporting File**: advs77817‐sup‐0001‐SuppMat.docx.

## Data Availability

The authors have nothing to report.
