# Supplementary material for: Sonogenetics Nanomedicine Activates the cGAS‐STING Pathway for Immunotherapy of Triple‐Negative Breast Cancer
Source: Adv Sci (Weinh). 2026 Sep 16:e77817. Online ahead of print. doi: 10.1002/advs.77817 (PMC13583103; doi:10.1002/advs.77817)
Supplement: Supplementary file 1 [file ADVS-9999-e77817-s001.docx]

*Supporting Information*

**Sonogenetics Nanomedicine Activates the cGAS-STING Pathway for Immunotherapy of Triple-Negative Breast Cancer**

Junya Song^1^, Yan Liu^1^, Shifeng Zhao^2^, Cheng Zhu^1, *^, Hongchang Guo^2, *^, Jin Chang^1, *^, Jun Kang^1, *^

1. School of Life Sciences, Faculty of Medicine, Tianjin University, Tianjin 300072, China.
2. Department of Cardiovascular Surgery, Beijing Anzhen Hospital, Capital Medical University, Beijing, China.

*Corresponding authors:

* Address correspondence to Jun Kang, jun.kang@tju.edu.cn; Jin Chang, jinchang@tju.edu.cn; Hongchang Guo, guohongchang@ccmu.edu.cn; Cheng Zhu, cheng_zhu@tju.edu.cn.

**Figure S1.** (a-d) Predicted binding models and BLI sensorgrams of miniprotein-2 to miniprotein-5 binding to HER3 protein at the indicated concentrations (625-5000 nM).

**Figure S2.** (a) Western blot analysis was performed to assess the levels of p-mTOR, mTOR, p-ERK, and ERK in 4T1 cells treated with HTIM, compared to the control group. (b) Analysis of phosphorylation levels of mTOR and ERK in 4T1 cells treated with HTIM (Mean ± SD, n = 4). The p values were calculated using one-way ANOVA; ****p < 0.0001.

**Figure S3.** (a) Visual appearance of HSMIN-LNP in deionized water, PBS, and RPMI 1640 at day 1 and day 7. (b) The encapsulation efficiency of HSMIN-LNP for 7 days (Mean ± SD, n = 4). (c) Responsive release profile of HTIM from HSMIN-LNP after ultrasound stimulation (Mean ± SD, n = 4).

**Figure S4.** (a) Representative confocal images showing the intracellular calcium ion levels in 4T1 cells under the indicated treatments in a calcium-free medium. Calcium ion levels (green: Cal-520) and nuclei (blue: Hoechst). Scale bar: 50 μm. (b) Quantification of mean fluorescence intensity (MFI) of Cal-520. (Mean ± SD, n = 3). The p values were calculated using one-way ANOVA; ns: not significant, **p < 0.01.

**Figure S5.** (a) Representative confocal images showing mtROS production in 4T1 cells. MtROS levels (red: MitoSox staining) and nuclei (blue: Hoechst). Scale bar: 50 μm. (b) Quantification of MFI of MitoSOX under the indicated treatment (Mean ± SD, n = 3). The p values were calculated using one-way ANOVA; ****p < 0.0001.

**Figure S6.** qPCR analysis of the relative mRNA expression levels of (a) CXCL9, (b) CXCL10, and (c) ISG15 in 4T1 cells (Mean ± SD, n = 4). The p values were calculated using one-way ANOVA; ***p < 0.001, ****p < 0.0001.

**Figure S7.** Representative bioluminescence images of subcutaneous 4T1 tumor-bearing mice after Luc-LNP administration, showing luciferase expression following luciferin injection over 16 days.

**Figure S8.** (a) Western blot analysis of TRPV4 protein expression in tumor tissues. (b) Quantitative analysis of changes in TRPV4 protein expression levels. (Mean ± SD, n = 3). The p values were calculated using one-way ANOVA; ***p < 0.001, ****p < 0.0001.

**Figure S9.** (a) H&E staining of heart, kidney, spleen, liver, lung from different groups. (b) Histological and immunofluorescent analysis of cardiac fibrosis.

**Figure S10.** (a-d) Blood biochemical indicator analysis (Mean ± SD, n = 3). The p values were calculated using one-way ANOVA; ns: not significant.

**Figure S11.** (a-h) Blood routine analysis (Mean ± SD, n = 3). The p values were calculated using one-way ANOVA; ns: not significant.

**Table S1. The optimized IL-15 gene sequence after comprehensive optimization of the codons of humans and mice.**

**Optimized IL-15 (5’-3’)**

AAGCTTATGAAGATCCTGAAGCCCTACATGAGAAACACAAGCATCAGCTGCTACCTGTGCTTCCTGCTCAACAGCCACTTCCTGACCGAGGCCGGCATCCACGTGTTCATCCTGGGCTGCGTGAGCGTGGGCCTGCCCAAGACCGAGGCCAACTGGATCGACGTGAGATACGACCTGGAGAAGATCGAGAGCCTGATTCAGAGCATCCACATCGACACCACCCTGTACACCGACAGCGACTTCCACCCTAGCTGCAAGGTGACCGCCATGAACTGCTTCCTGCTGGAGCTGCAAGTGATCCTGCACGAGTACAGCAACATGACCCTGAACGAGACCGTGAGAAACGTGCTGTACCTGGCCAACAGCACCCTGAGCAGCAACAAGAACGTGGCCGAGAGCGGCTGCAAGGAGTGCGAGGAGCTGGAGGAGAAGACCTTCACCGAGTTCCTGCAGAGCTTCATCAGAATCGTGCAGATGTTCATCAACACAAGCTGAGGCCGGCC

**Table S2. The NFAT-responsive IL-15 expression plasmid system labeled with mCherry fluorescent tag (containing the sequence segment from NFAT-RE to mCherry).**

**pGL4.30[NFAT-RE-IL15-P2A-mCherry-Hygro] (5’-3’)**

AGCGGAGGAAAAACTGTTTCATACAGAAGGCGTGGAGGAAAAACTGTTTCATACAGAAGGCGTGGAGGAAAAACTGTTTCATACAGAAGGCGTAGATCTAGACTCTAGAGGGTATATAATGGAAGCTCGAATTCCAGCTTGGCATTCCGGTACTGTTGGTAAAGCCACCAAGCTTATGAAGATCCTGAAGCCCTACATGAGAAACACAAGCATCAGCTGCTACCTGTGCTTCCTGCTCAACAGCCACTTCCTGACCGAGGCCGGCATCCACGTGTTCATCCTGGGCTGCGTGAGCGTGGGCCTGCCCAAGACCGAGGCCAACTGGATCGACGTGAGATACGACCTGGAGAAGATCGAGAGCCTGATTCAGAGCATCCACATCGACACCACCCTGTACACCGACAGCGACTTCCACCCTAGCTGCAAGGTGACCGCCATGAACTGCTTCCTGCTGGAGCTGCAAGTGATCCTGCACGAGTACAGCAACATGACCCTGAACGAGACCGTGAGAAACGTGCTGTACCTGGCCAACAGCACCCTGAGCAGCAACAAGAACGTGGCCGAGAGCGGCTGCAAGGAGTGCGAGGAGCTGGAGGAGAAGACCTTCACCGAGTTCCTGCAGAGCTTCATCAGAATCGTGCAGATGTTCATCAACACAAGCTGAGGCCGGCCGCCACCAATTTCAGCCTGCTGAAGCAAGCCGGCGACGTCGAAGAGAACCCTGGCCCTGAATTCGGATCCGCCACCATGGTGAGCAAGGGCGAGGAGGATAACATGGCCATCATCAAGGAGTTCATGCGCTTCAAGGTGCACATGGAGGGCTCCGTGAACGGCCACGAGTTCGAGATCGAGGGCGAGGGCGAGGGCCGCCCCTACGAGGGCACCCAGACCGCCAAGCTGAAGGTGACCAAGGGTGGCCCCCTGCCCTTCGCCTGGGACATCCTGTCCCCTCAGTTCATGTACGGCTCCAAGGCCTACGTGAAGCACCCCGCCGACATCCCCGACTACTTGAAGCTGTCCTTCCCCGAGGGCTTCAAGTGGGAGCGCGTGATGAACTTCGAGGACGGCGGCGTGGTGACCGTGACCCAGGACTCCTCCCTGCAGGACGGCGAGTTCATCTACAAGGTGAAGCTGCGCGGCACCAACTTCCCCTCCGACGGCCCCGTAATGCAGAAGAAGACCATGGGCTGGGAGGCCTCCTCCGAGCGGATGTACCCCGAGGACGGCGCCCTGAAGGGCGAGATCAAGCAGAGGCTGAAGCTGAAGGACGGCGGCCACTACGACGCTGAGGTCAAGACCACCTACAAGGCCAAGAAGCCCGTGCAGCTGCCCGGCGCCTACAACGTCAACATCAAGTTGGACATCACCTCCCACAACGAGGACTACACCATCGTGGAACAGTACGAACGCGCCGAGGGCCGCCACTCCACCGGCGGCATGGACGAGCTGTACAAGTAA

Purple: 3NFAT-RE; green: minP; yellow: kozak sequence; blue: optimized IL-15 sequence; orange: P2A sequence; red: mCherry.

**Table S3. Correspondence between driving voltage (Vpp), calibrated acoustic power (P_CW_), and time-averaged acoustic power (P_TA_).**

| Driving voltage (Vpp) | Transducer1  P_CW_ (mW) | Transducer2  P_CW_ (mW) | Average  P_CW_ (mW) | P_TA_ (mW)  (=P_CW_*DC) |
| --- | --- | --- | --- | --- |
| 20 | 112 | 100 | 106 | 21 |
| 40 | 364 | 382 | 373 | 74 |
| 60 | 805 | 856 | 831 | 166 |
| 80 | 1467 | 1522 | 1495 | 299 |
| 100 | 2306 | 2335 | 2321 | 464 |
| 120 | 3347 | 3350 | 3349 | 670 |

**Table S4. Primer sequences**

| **Gene name** | **Forward primer** | **Reverse primer** |
| --- | --- | --- |
| Actb | TGAGCTGCGTTTTACACCCT | GCCTTCACCGTTCCAGTTTT |
| Ifnb1 | GTCCTCAACTGCTCTCCACT | CCTGCAACCACCACTCATTC |
| TNFα | AGGCTGCCCCGACTACGT | GACTTTCTCCTGGTATGAGATAGCAAA |
| Il6 | ACAAGTCGGAGGCTTAATTACACAT | TTGCCATTGCACAACTCTTTTC |
| Il1b | TCGCTCAGGGTCACAAGAAA | CATCAGAGGCAAGGAGGAAAA |
| Cxcl9 | TCATTGCTACACTGAAGAACGGAG | ACGACGACGACTTTGGGGTG |
| Cxcl10 | ATCATCCCTGCGAGCCTATCCT | GACCTTTTTTGGCTAAACGCTTTC |
| Isg15 | AAGAAGCAGATTGCCCAGAA | TCTGCGTCAGAAAGACCTCA |

**Table S5. Antibodies for flow cytometry**

| **Antibody** | **Source** | **Cat Number** | **Application** |
| --- | --- | --- | --- |
| PE/Cyanine7 anti-mouse CD45 | Biolegend | 147704 | Flow Cytometry |
| FITC anti-mouse CD3 | Biolegend | 100204 | Flow Cytometry |
| APC anti-mouse CD4 | Biolegend | 100412 | Flow Cytometry |
| PE anti-mouse CD8a | Biolegend | 100708 | Flow Cytometry |
| PE anti-mouse CD11c | Biolegend | 117308 | Flow Cytometry |
| APC anti-mouse CD80 | Biolegend | 104714 | Flow Cytometry |
| FITC anti-mouse CD86 | Biolegend | 105006 | Flow Cytometry |
| Pacific Blue-CD45 | Biolegend | 157212 | Flow Cytometry |
| APC anti-mouse CD49b | Biolegend | 103515 | Flow Cytometry |
| Pacific Blue anti-mouse CD44 | Biolegend | 156005 | Flow Cytometry |
